# Joint species movement modeling: how do traits influence movements?

**DOI:** 10.1002/ecy.2622

**Published:** 2019-02-21

**Authors:** Otso Ovaskainen, Danielle Leal Ramos, Eleanor M. Slade, Thomas Merckx, Gleb Tikhonov, Juho Pennanen, Marco Aurélio Pizo, Milton Cezar Ribeiro, Juan Manuel Morales

**Affiliations:** ^1^ Organismal and Evolutionary Biology Research Programme University of Helsinki P.O. Box 65 Helsinki 00014 Finland; ^2^ Department of Biology Centre for Biodiversity Dynamics Norwegian University of Science and Technology Trondheim N‐7491 Norway; ^3^ Departamento de Ecologia Instituto de Biociências Universidade Estadual Paulista (UNESP) Rio Claro Sao Paulo Brazil; ^4^ Department of Zoology University of Oxford South Parks Road Oxford OX1 3PS United Kingdom; ^5^ Behavioural Ecology and Conservation Group Biodiversity Research Centre, Earth and Life Institute UCLouvain Croix du Sud 4‐5, bte L7.07.04 Louvain‐la‐Neuve BE‐1348 Belgium; ^6^ Departamento de Zoologia Instituto de Biociências Universidade Estadual Paulista (Unesp) Rio Claro Sao Paulo Brazil; ^7^ Grupo de Ecología Cuantitativa INIBIOMA‐CRUB CONICET Avenida Pioneros 2350, S.C. de Bariloche Río Negro Argentina

**Keywords:** birds, community model, hierarchical model, joint species model, moths, movement model, statistical model

## Abstract

Joint species distribution modeling has enabled researchers to move from species‐level to community‐level analyses, leading to statistically more efficient and ecologically more informative use of data. Here, we propose joint species movement modeling (JSMM) as an analogous approach that enables inferring both species‐ and community‐level movement parameters from multispecies movement data. The species‐level movement parameters are modeled as a function of species traits and phylogenetic relationships, allowing one to ask how species traits influence movements, and whether phylogenetically related species are similar in their movement behavior. We illustrate the modeling framework with two contrasting case studies: a stochastic redistribution model for direct observations of bird movements and a spatially structured diffusion model for capture–recapture data on moth movements. In both cases, the JSMM identified several traits that explain differences in movement behavior among species, such as movement rate increasing with body size in both birds and moths. We show with simulations that the JSMM approach increases precision of species‐specific parameter estimates by borrowing information from other species that are closely related or have similar traits. The JSMM framework is applicable for many kinds of data, and it facilitates a mechanistic understanding of the causes and consequences of interspecific variation in movement behavior.

## Introduction

Ecologists are increasingly recognizing that ecological and evolutionary processes are structured across many kinds of hierarchical levels, as reflected by the increasing popularity of hierarchical Bayesian modeling (Parent and Rivot 2012, Gimenez et al. 2014). For example, joint species distribution modeling (JSDM) is revolutionizing the field of statistical community ecology, as it allows species‐ and community‐level inference to be simultaneously derived by integrating data on species occurrences, environmental covariates, species traits, and their phylogenetic relationships (Warton et al. 2015, Ovaskainen et al. 2017). In movement ecology, the need for hierarchical modeling arises from variation in movement characteristics expressed within individuals, among individuals, and among species, as well as from the need to account for observation and measurement error (Patterson et al. 2008). The movement characteristics of an individual may vary over space, e.g., depending on the habitat type, or over time, e.g., depending on the prevailing weather conditions, reproductive phase, or temporal variation in the individual's motivation to move. Such variation has been accounted for in statistical models by assuming that the movement characteristics depend on the measured environmental parameters (Morales et al. 2004, Ovaskainen et al. 2008), or by modeling switches among different movement modes, e.g., via hidden Markov models (Morales et al. 2004, Gurarie et al. 2009, Langrock et al. 2012). Movement characteristics also vary among individuals, and these have been accounted for either by fitting a movement model to each individual separately and then making population‐level inferences in a separate second step (Hooten et al. 2016), or by adding random effects to parameters governing, e.g., step lengths and/or turning angles (Langrock et al. 2012, Hopcraft et al. 2014, Hooten et al. 2017).

Variation in movement characteristics among species has typically been analysed in a post hoc manner using a two‐step approach: (1) fitting a movement model separately for each species; (2) comparing the model structures or parameter estimates among the species (Morales et al. 2013). This two‐step approach is likely to lead to compromised statistical efficiency especially in the analysis of sparse data. Moreover, this approach does not allow shared traits between species to be incorporated into the models. Here, we borrow from recent developments in JSDM (Warton et al. 2015, Ovaskainen et al. 2017) to build a joint species movement model (JSMM) that enables statistically efficient use of multispecies movement data. In species distribution modeling, regression approaches are commonly used to describe how species occurrence depends on environmental covariates (Elith and Leathwick 2009). JSDMs can greatly facilitate a more accurate estimation of such parameters, especially for rare species with sparse data, by incorporating hierarchical layers that model shared responses to environmental covariates among the species (Ovaskainen and Soininen 2011). Such models can be extended to give community‐level inference by assuming that the responses of the species to their environment depend on their traits and/or phylogenetic relationships (Ovaskainen et al. 2017). Here, we transfer these ideas into movement ecology, by building a JSMM that models species‐specific movement parameters as a function of species traits, phylogenetic relationships, or both.

## Materials and Methods

### The data

While our approach for the joint estimation of species‐ and community‐level movement parameters applies to many kinds of movement models and data, here we illustrate the approach with two contrasting data sets: direct observations of bird movements and spatial capture–recapture data on moth movements.

### Direct observations on bird movements

**Figure 1:**
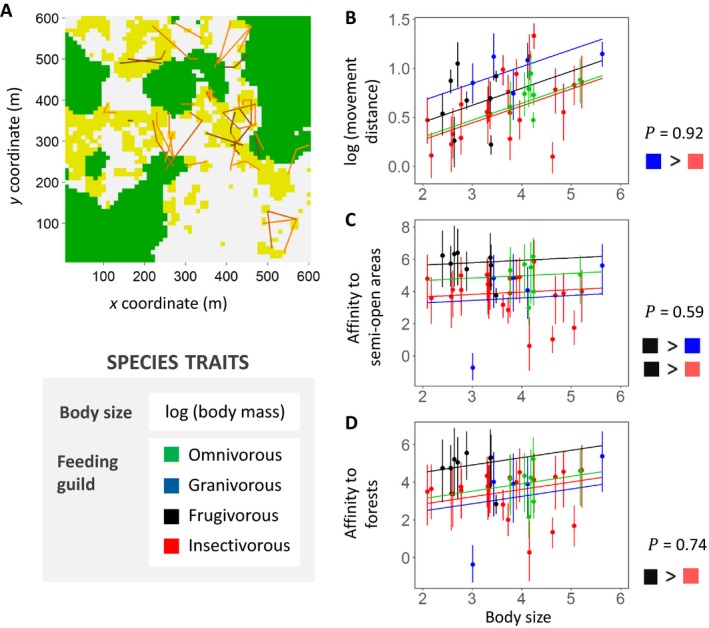
The bird case study. Panel A shows a map of the study area (green, forest; yellow, semi‐open habitat; gray, open habitat) and an example of the movement data for *Tangara sayaca* (Sayaca Tanager), where differently colored lines correspond to different individuals. Panels B, C, and D show, for each movement parameter, the species‐specific parameter estimates (the dots show the posterior mean and error bars are 95% credible intervals) and the expected movement parameter based on species traits (the lines show the posterior mean). For the pairs of feeding guilds shown as [color 1] > [color 2], the posterior probability for feeding class corresponding to color 1 having a higher parameter value than the feeding class corresponding to color 2 (measured as the difference in the ζ parameters being positive) was at least 0.85. The posterior probability *P* by which each movement parameter increases with body size is shown for each panel.

The bird movement data are based on binocular observations, with the method adapted from Morales et al. (2013). The study area is a 600 × 600 m square landscape with ~40% of arboreal cover, within the Brazilian Atlantic Forest biodiversity hotspot (Fig. 1A). We divided this landscape into a regular 10 × 10 m grid with 3,600 cells, and classified the grid cells into three habitat types: (1) forest habitat, (2) open habitat (mainly pasture), and (3) semi‐open habitat (narrow corridors of trees, single trees and small groups of trees outside forest patches). We collected data on 43 bird species from September to December of 2014 and 2015, during 72 h of observation. The locations of the birds were recorded at the level of the grid cell by comparing the observed location to a grid overlaid on a high‐resolution Google Maps image. We considered the movements as discrete steps in which the bird left one location where it had been stationary and flew to another location, within the same grid cell or to one or several grid cells away. We did not consider the temporal dimension in this analysis, i.e., when the bird makes the next move, or how long it takes to perform each move. The recorded tracks were short sequences of steps (one to seven steps) and the same bird individual was only rarely observed for more than one sequence of steps.

For species traits, we acquired information on body mass and feeding guild. Birds were classified into four feeding guilds, based on Wilman et al. (2014) and on our own experience: granivorous, frugivorous, insectivorous, or omnivorous birds. For trait values, see Appendix S3: Table S1. We constructed a phylogenetic correlation matrix **C** from the mean phylogenetic tree provided by Jetz et al. (2014) with the package ape 4.0 (Paradis et al. 2004) by assuming the diffusion model of trait evolution, and thus defining correlations between two species as the proportion of shared evolutionary history.

### Spatial capture–recapture data on moth movements

**Figure 2:**
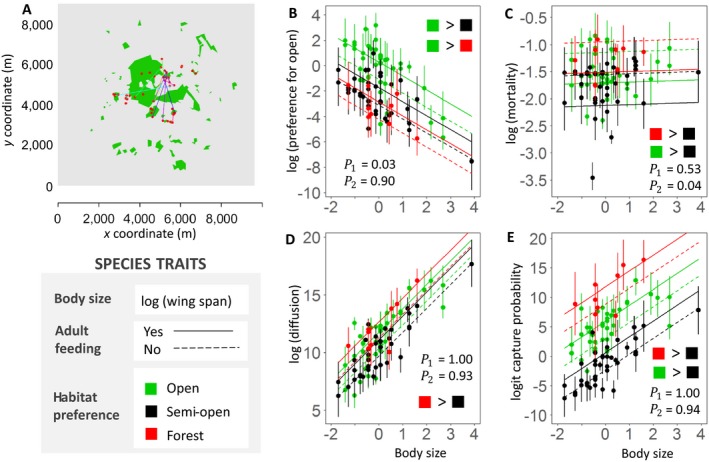
The moth case study. Panel A shows a map of the study area (green, forest; gray, open habitat), the locations of the 44 light traps (red dots), and an example of the movement data for *Apamea lithoxylaea* (Light Arches), with differently colored lines corresponding to different individuals. Panels B, C, D, and E show, for each movement parameter, the species‐specific parameter estimates (the dots show the posterior mean and error bars are 95% credible intervals) and the expected movement parameter based on species traits (the lines show the posterior mean, with solid lines corresponding to species that feed as adults and dashed lines to species that do not feed as adults). For the pairs of forest affinity classes shown in the panels as [color 1] > [color 2], the posterior probability for forest affinity corresponding to color 1 having a higher parameter value than the forest affinity corresponding to color 2 (measured as the difference in the ζ parameters being positive) was at least 0.85. The posterior probabilities by which each movement parameter increases with body size (*P*_1_) or adult feeding (*P*_2_) are shown in the panels.

The moth movement data are based on a spatial capture–recapture study. Data collection was carried out within a fragmented landscape consisting of forest patches within agricultural open habitats in Southern England (Fig. 2A). Forty‐four actinic 6W Heath light traps (Watkins & Doncaster, Leominster, UK) were set up in fixed locations in forest patches and at solitary oak trees (either isolated or within a hedgerow) within the agricultural matrix. The average distance among all pairwise trap combinations was 1,739 ± 20 m (mean ± SE; maximum 4,131 m; minimum 45 m). The study ran from 14 June until 24 July 2009 during which traps were operational for 31 nights. The active traps alternated in two sets so that on each night about one‐half of the traps were activated, and these were checked each morning. Actinic 6W traps have been observed to attract moths from distances below 30 m (Merckx and Slade 2014) and thus are not expected to influence the landscape‐level movement patterns.

We included in this study 87 macro‐moth species that occur in forest landscapes, for which the flight periods coincide with the study period, and which are sufficiently common, easy to identify, and easy to mark. The individuals were marked at first capture by writing a unique number on the left forewing. After marking, moths were released at the place of capture into nearby tall vegetation. All 31 nights met the sampling criteria: minimum night temperature was at least 10°C, maximum wind speed was at most 20 km/h, and maximum precipitation risk was at most 50%. For more details on the empirical study, see Slade et al. (2013).

We recorded the following traits for each species based on Manley (2008) and Waring and Townsend (2009): habitat preference (classified as open, semi‐open, or forest), log‐transformed wingspan (mm; normalized to mean 0 and variance 1), and whether the species feeds as an adult or not. For trait values, see Appendix S3: Table S2. As there is no sound phylogeny yet for Eurasian moths (Betzholtz and Franzén 2011), we used as a simple proxy a taxonomical tree, where we assumed equal branch lengths for the levels of species, genus, subfamily, and family. We constructed a taxonomical correlation matrix **C** from the taxonomic tree using the same procedure as described in the bird case study.

### The joint species movement model

We first describe the general JSMM, and then describe how we applied it specifically to the two data sets described above. We denote the number of species by *n*_*s*_, the number of species traits by *n*_*t*_ and the number of species‐specific movement parameters by *n*_*p*_. We combine the species‐specific movement parameters into the *n*_*s*_ × *n*_*p*_ matrix **Θ**, so that each row of **Θ**, i.e., the vector **Θ**_*s*_, contains the parameters for species *s*. The number of the parameters (*n*_*p*_) and their interpretations depend on the specific movement model. We assume that, possibly after transformations, the parameters can obtain any real values. This assumption is necessary as we will model the matrix **Θ** with a multivariate normal distribution.

To determine how the movement characteristics of the species (as described by the parameters **Θ**) depend on their traits and phylogenetic relationships, we vectorize the matrix **Θ** to the *n*_*s*_ *n*_*p*_ × 1 vector **θ **= vec(**Θ**), and model it using a multivariate normal distribution as

(1) $$\theta ∼N\left(m,\Sigma ⊗\left[\rho C+\left(1-\rho \right)I_{n_{s}}\right]\right).$$

here the *n*_*s*_ *n*_*p*_ **× 1 vector **m **= vec(**M**) is the vectorized version of the *n*_*s*_ × *n*_*p*_ matrix **M** (with elements *m*_*sp*_, where *p* = 1, …, *n*_*p*_ is the index for the movement parameter), and it gives the expected movement parameters based on species traits. The influence of traits is modeled with a linear regression model $m_{\mathrm{sp}}=\sum_{k}t_{\mathrm{sk}}\zeta_{\mathrm{kp}}$, where the index *k* runs over the *n*_*t*_ traits, *t*_*sk*_ is the trait *k* for species *s*, and the parameter ζ_*kp*_ measures the influence of trait *k* on movement parameter *p*. Arranging the regression parameters ζ_*kp*_ into a *n*_*t*_ × *n*_*p*_ matrix **Z** and the trait values $t_{\mathrm{sk}}$ into a *n*_*s*_ × *n*_*t*_ matrix **T**, we can write in matrix form **M** = **TZ**. To include the intercept into the model, we set *t*_*s*1_ = 1 for all species *s*. In the absence of trait information, only the intercept is included, in which case the expectation **M**_*s*·_ is the same for all species. The *n*_*p*_ × *n*_*p*_ variance–covariance matrix **Σ** in Eq. (1) models the species‐specific deviations around the expectation based on species traits, and ⨂ is the Kronecker (outer) product. The *n*_*s*_ × *n*_*s*_ matrix **C** is a phylogenetic correlation matrix that can be derived from a phylogenetic tree based on genetic data (as we did in the bird case study), or constructed from taxonomic information if a quantitative phylogeny is not available (as we did with the moth case study). The matrix $I_{n_{s}}$ is the identity matrix of dimension *n*_*s*_, and the parameter 0 ≤ ρ ≤ 1 measures the strength of the phylogenetic signal.

To fit the model to the data with Bayesian inference, we developed a Markov chain Monte Carlo (MCMC) sampling scheme that uses a Metropolis‐Hastings step to sample the species‐specific parameters **Θ**, whereas the parameters **Z**,** Σ,** and ρ are sampled directly from their full conditional distributions (for technical details and prior distributions, see Appendix S1, and for MCMC convergence, see Appendix S3). We tested the validity of the estimation scheme by generating replicated simulated data sets of various sizes and types (including nonlinear relationships between species traits and movement parameters) and examining whether the parameter estimates converged to the true values as the data size increased (see Appendix S2). In the Appendix S2, we further illustrate how posterior predictive checking can be used to test the structural model assumptions. R code used to fit the model is available (see *Data Availability*).

To be able to apply the JSMM to a particular data type, the user needs to define $p\left(y_{s}|\Theta_{s\cdot }\right)$, i.e., the likelihood of observing the data *y*_*s*_ for species *s*, given the parameter vector **Θ**_*s*·_. We next describe the likelihood functions that we assumed in the bird and the moth case studies.

### Stochastic redistribution model for bird movements

The bird data consist on average of 12 (min = 1, max = 109) movement steps for *n*_*s*_ = 43 species. Assuming that an individual of species *s* is currently at grid cell *i*, we model the probability *p*_*sji*_ that it will next move to grid cell *j* by

(2) $$p_{\mathrm{sji}}=K_{\mathrm{is}}\text{exp}\left(-d_{\mathrm{ij}}/\alpha_{s}\right)\text{exp}\left(\beta_{s}^{\left(1\right)}h_{j}^{\left(1\right)}\right)\text{exp}\left(\beta_{s}^{\left(2\right)}h_{j}^{\left(2\right)}\right).$$

In Eq. (2), *d*_*ij*_ is the distance between the grid cells *i* and *j*, and the parameter α_*s*_ > 0 models the characteristic step length of species *s*. The variable $h_{j}^{\left(1\right)}\in \left\{0,1\right\}$ (respectively, $h_{j}^{\left(2\right)}$) is an indicator of whether grid cell *j* belongs to the semi‐open habitat (respectively, forest) and the parameter $\beta_{s}^{\left(1\right)}$ (respectively, $\beta_{s}^{\left(2\right)}$) measures the affinity of the species to semi‐open (respectively, forest) habitats compared to open habitat. The normalizing constant *K*_*is*_ is defined so that the probabilities sum to unity over the target cells, i.e., that $\sum_{j}p_{\mathrm{sji}}=1$ for all *i* and *s*. The *n*_*p*_ = 3 species‐specific parameters form the vector

(3) $$\Theta_{s\cdot }=\left(\log\left(\alpha_{s}\right)\;\beta_{s}^{\left(1\right)}\;\beta_{s}^{\left(2\right)}\right).$$

The likelihood $p\left(y_{s}|\Theta_{s\cdot }\right)$ for observing the movement data *y*_*s*_ was computed as the product of the single‐step movement probabilities given by Eq. (2).

### Spatially structured diffusion model for moth movements

The moth data consist on average of 149.6 (minimum = 1, maximum = 1316) marked individuals and 8.1 (minimum = 0, maximum = 166) recaptures for *n*_*s*_ = 87 species. We model the individual‐level movements by diffusion, which can be considered as an approximation of a random walk (Turchin 1998), supplemented with habitat selection (also called edge‐mediated behavior; Schultz and Crone 2001) at edges between forests and open areas. The parameters of the movement model, for species *s*, are the diffusion coefficient *D*_*s*_ (unit m^2^/d) measuring the movement rate, the mortality rate *m*_*s*_ (unit 1/d), and the habitat selection parameter *k*_*s*_, measuring the relative attractiveness of open areas over forest (Ovaskainen 2008). We assumed for simplicity that both the movement rate and the mortality rate are the same for forests and open areas.

An observation model was used to describe the trapping process. We assume that once a trap was active over a one‐night period, it attracted and captured individuals of species *s* with probability *q*_*s*_ from a circular area within a 30 m radius around the trap. We note that an identical capture probability can be obtained by either a large attraction radius and a low capture efficiency or a small attraction radius and a high capture efficiency, and our data do not allow the separation of these two parameters. The *n*_*p*_ = 4 species‐specific parameters form the vector

(4) $$\Theta_{s\cdot }=\left(\text{log}\left(D_{s}\right)\;\text{log}\left(k_{s}\right)\;\text{log}\left(m_{s}\right)\;\text{logit}\left(q_{s}\right)\right).$$

The likelihood $p\left(y_{s}|\Theta_{s\cdot }\right)$ for observing the movement data *y*_*s*_ was computed as in Ovaskainen (2004), i.e., by triangulating the landscape and using a finite‐element method to solve the time‐evolution for the probability density for the individuals' location under the diffusion model.

## Results

The performance of the estimation scheme was successfully validated with simulated data as shown by the posterior distributions becoming increasingly concentrated around the true values with an increasing amount of data (Appendix S2). As expected, the accurate estimation of the community‐level parameters requires data on many species, whereas the accurate estimation of species‐specific parameters requires many movement steps for the focal species (Appendix S2). A comparison between the JSMM and individually parameterized single‐species models further shows that borrowing information from other species increases the precision of species‐specific parameter estimates (Appendix S2).

The parameter estimates of the bird model show that generally all birds and especially frugivorous birds prefer semi‐open and forest habitats over the open habitats (Fig. 1C, D shows positive parameter values). Movement distances increase with body size, and granivorous birds move on average larger distances than birds from the other feeding guilds (Fig. 1B). The parameter estimates of the moth model show that movement rate and capture probability increase with body size, and for species that feed as adults (Fig. 2D, E), whereas preference for open areas decreases with body size, but is higher for adult feeders (Fig. 2B). Mortality was lower for species that feed as adults than for species that do not feed as adults, and for species that prefer semi‐open areas, but was not affected by body size (Fig. 2C). Movement rate and capture probability were highest for species that prefer forests (Fig. 2D, E).

We did not find evidence for a phylogenetic signal in the movement parameters, as the posterior probability for ρ > 0 was 0.50 for the bird and 0.41 for the moth case study.

## Discussion

The increasing interest in movement ecology, fueled by methodological advances in data collection and analysis, is promoting an unprecedented availability of movement data on individuals and species in numerous environmental contexts (Nathan et al. 2008, Kranstauber et al. 2011, Kays et al. 2015). We developed the JSMM framework for statistically efficient estimation of movement parameters as a function of species traits and phylogenetic relationships. This approach is promising for answering many kinds of questions in movement ecology, and in particular to tackle the challenge on how to best use the large amount of sparse data that is currently accumulating in various databases (Kranstauber et al. 2011).

Previous research assessing how functional traits affect movement (Nieminen et al. 1999, Spiegel and Nathan 2007, Sekar 2012, Stevens et al. 2012, 2014, Neuschulz et al. 2013) has been based on two‐step analyses where each species is first modeled separately. As species‐specific models require lots of data, these analyses have been restricted to the few most abundant species only. In contrast, our approach allows the inclusion of a large number of species, also those for which even a single movement step is observed. The results from our case studies showed that both bird and moth movements can, to a large extent, be related to species traits. Our finding of movement rate increasing with body size is in line with earlier research both for birds (Spiegel and Nathan 2007, Neuschulz et al. 2013) and Lepidoptera (Nieminen et al. 1999, Beck and Kitching 2007, Öckinger et al. 2010, Sekar 2012, Stevens et al. 2012). Besides moving larger distances, large birds and moths showed lower affinity to open habitat, possibly because such habitat makes large species more detectable to predators. In moths, species with a strong affinity to forest moved larger distances, which may be a result of these forest specialists being exposed to stronger selection pressures for increased dispersal ability due to the long history of forest fragmentation in the UK. Their high dispersal ability coupled with their strong affinity for forest habitats makes this group particularly vulnerable to further habitat fragmentation and particularly reliant on connectivity between forest patches (Slade et al. 2013). In moths, we found the capability of adult feeding to be linked to a lower mortality, higher movement rates, and a higher preference for open habitat. Adult feeders may need to be more mobile to locate enough resources, and previous work on hawkmoths has also found that adult feeders are longer lived, and therefore move further and are less habitat specific (Beck and Kitching 2007).

In our results, the phylogenetic relationships turned out not to be strongly related to movement. In moths, the lack of phylogenetic signal is in agreement with previous findings on noctuid and sphingid moths (Nieminen et al. 1999, Beck and Kitching 2007) and with meta‐analyses on butterflies (Sekar 2012, Stevens et al. 2012), suggesting movement rate is an evolutionarily labile character, mainly dependent on species‐specific ecological and life‐cycle characteristics. In birds, the lack of phylogenetic signal is probably because diet can vary substantially among closely related species (Wilman et al. 2014), and because diet strongly influences movement patterns. Granivorous birds, for instance, feed on the seeds of herbaceous plants growing in open areas, explaining their high affinity to open habitats. Also, frugivorous and granivorous birds do not maintain territories (Neuschulz et al. 2013), possibly explaining why we found them to move larger distances than insectivores.

We applied the JSMM approach to two contrasting case studies to illustrate that it is applicable to almost any kind of multispecies movement data, such as GPS‐based tracking data (Reisinger et al. 2018), spatial capture–recapture data based on bird ringing (Paradis et al. 1998), or marking of insects (Scriven et al. 2017), trapping of small mammals (Puttker et al. 2012), or camera‐trapping and noninvasive genetic sampling (Royle et al. 2018). The JSMM approach can be applied to any likelihood‐based analysis of multispecies movement data by adding a hierarchical layer that models species‐specific parameters as a function of their traits and phylogenetic relationships (Eq. (1)). The model can involve any kind of movement parameters, such as the level of temporal autocorrelation, home‐range size, or tendency to return to familiar sites, to name a few. Studying movement at the community level helps to relate data with theory, such as the movement ecology paradigm (Nathan et al. 2008). For example, hypothesized proxies of species‐specific navigation and motion capacities can be incorporated into the analyses as species traits to statistically examine their influence to observed movement behavior. We note that as with any modeling exercise, comprehensive validation of structural model assumptions should be done when applying the JSMM framework to a specific case study. To illustrate how this can be done in practice, we discuss the general principle of posterior predictive checking in the context of the bird case study in Appendix S2.

The movements of organisms greatly influence which individuals, species, and environmental factors they will interact with and, consequently, affect both their ecological and evolutionary dynamics (Nathan et al. 2008, Morales et al. 2010, Jeltsch et al. 2013). Thus, identifying how species traits influence organismal movement is crucial for building links from movement ecology to population and evolutionary ecology, and for assessing the consequences of movement on ecosystem functioning and management. We thus expect that the JSMM framework developed here will find applications not only in basic ecological research, but also in biodiversity conservation projects.

## Data Availability

Associated R code is available on Zenodo: https://doi.org/10.5281/zenodo.2530320.
