# Supplementary material for: Joint species movement modeling: how do traits influence movements?
Source: Ecology. 2019 Feb 21;100(4):e02622. doi: 10.1002/ecy.2622 (PMC6850360; doi:10.1002/ecy.2622)
Supplement: Supplementary file 1 [file ECY-100-na-s001.pdf]

Supporting Information. Otso Ovaskainen, Danielle Leal Ramos, Eleanor M. Slade, Thomas Merckx, Gleb Tikhonov, Juho Pennanen, Marco Aurélio Pizo, Milton Cezar Ribeiro, and Juan Manuel Morales. 2019. Joint species movement modeling: how do traits influence movements? *Ecology.*

Appendix S1. Prior distributions and posterior sampling scheme

Prior distributions

As described in the main text, the model to be fitted is

$$\boldsymbol{\theta} \sim \mathrm{N}\left(\mathbf{m}, \boldsymbol{\Sigma} \otimes \left[\rho \mathbf{C} + (1-\rho)\mathbf{I}_{n_s}\right]\right),$$

where the likelihood of the movement data depends on the parameters $\boldsymbol{\Theta}$. The mean $\mathbf{m} = \mathrm{vec}(\mathbf{TZ})$ is a vectorized version of the matrix $\mathbf{M} = \mathbf{TZ}$, where the matrix $\mathbf{T}$ consists of the elements t_{sk}. We denote by $\mathbf{z} = \mathrm{vec}(\mathbf{Z})$ the vectorization of the matrix $\mathbf{Z}$.

The parameters for which prior distributions need to be defined are $\mathbf{z}$, $\boldsymbol{\Sigma}$ and ρ.

- For the vector $\mathbf{z}$**,** we assumed the multivariate normal prior $\mathbf{z} \sim \mathrm{N}(\boldsymbol{\mu}_{\mathrm{z}}, \boldsymbol{\Sigma}_{\mathrm{z}})$, where we set the mean $\boldsymbol{\mu}_{\mathrm{z}}$ to a zero vector and the variance-covariance matrix $\boldsymbol{\Sigma}_{\mathrm{z}}$ to an identity matrix. We note that as the mean is set to zero, we do not make any a priori assumptions about directions of habitat preferences.
- For $\boldsymbol{\Sigma}$ we assumed an Inverse-Wishart prior $\mathrm{W}^{-1}(\boldsymbol{\Psi}, \nu)$, where we set the degrees of freedom to the number of parameters ($\nu = n_p$) and the scale matrix $\boldsymbol{\Psi}$ to the identity matrix.
- For the phylogenetic signal parameter ρ, we assumed a discrete prior, which assigned the probability of 0.5 for $\rho = 0$ (corresponding to independence among species), and the remaining probability of 0.5 uniformly to the range (0,1], discretised to 100 values.

Posterior sampling by MCMC

To fit the model to the data, we developed a Markov chain Monte Carlo (MCMC) sampling scheme in which we updated each of the following parameters in turn: $\boldsymbol{\theta}$, $\mathbf{z} = \mathrm{vec}(\mathbf{Z})$, $\boldsymbol{\Sigma}$ and ρ.

- The species-specific parameters $\boldsymbol{\theta}$ were sampled one at a time using a Metropolis-Hastings step in which the proposal distribution was adapted during the burn-in to reach an optimal acceptance ratio of 0.44. Additionally, in order to avoid slow MCMC mixing due to posterior correlations, during the burn-in we adaptively rotated the proposal space to align with the eigenvectors of the posterior distribution sampled thus far.

- The parameter $\mathbf{z}$ was sampled directly from its full conditional which is $\mathbf{z} \sim \mathrm{N}(\boldsymbol{\mu}_{\mathrm{z}*}, \boldsymbol{\Sigma}_{z*})$, where

$$\boldsymbol{\Sigma}_{z*} = \left(\boldsymbol{\Sigma}_z^{-1} + \mathbf{X}^{\mathrm{T}}(\boldsymbol{\Sigma} \otimes \mathbf{W})^{-1}\mathbf{X}\right)^{-1},$$
$$\boldsymbol{\mu}_{\mathrm{z}*} = \boldsymbol{\Sigma}_{z*}\left(\boldsymbol{\Sigma}_z^{-1}\boldsymbol{\mu}_{\mathrm{z}} + \mathbf{X}^{\mathrm{T}}(\boldsymbol{\Sigma} \otimes \mathbf{W})^{-1}\boldsymbol{\theta}\right),$$
$$\mathbf{X} = \mathbf{I}_{n_p} \otimes \mathbf{T},$$
$$\mathbf{W} = \left[\rho \mathbf{C} + (1-\rho)\mathbf{I}_{n_s}\right].$$

- The parameter $\boldsymbol{\Sigma}$ was sampled directly from its full conditional $W^{-1}(\boldsymbol{\Psi}_*, \nu_*)$, where

$$\nu_* = \nu + n_s,$$
$$\boldsymbol{\Psi}_* = \boldsymbol{\Psi} + \mathbf{A},$$
$$\mathbf{A} = (\boldsymbol{\Theta} - \mathbf{M})^{\mathbf{T}}\mathbf{W}^{-1}(\boldsymbol{\Theta} - \mathbf{M}),$$
$$.$$

- The parameter ρ was sampled directly from its full conditional by using a discrete grid sampler. By Eq. 2, the density of the full conditional for ρ follows

$$p(\rho|\boldsymbol{\theta}) \propto p(\rho)(\boldsymbol{\Sigma}\otimes\mathbf{W})^{-\frac{1}{2}} \exp\left(-(\boldsymbol{\theta}-\mathbf{m})^{\mathbf{T}}(\boldsymbol{\Sigma}\otimes\mathbf{W})^{-1}(\boldsymbol{\theta}-\mathbf{m})/2\right).$$

To increase computational speed, in the beginning of the MCMC approach, we pre-computed the determinants and inverses of the variance-covariance matrices $\mathbf{W}$ for all ρ values included in the discrete grid prior (see above).
