# Supplementary material for: Joint species movement modeling: how do traits influence movements?
Source: Ecology. 2019 Feb 21;100(4):e02622. doi: 10.1002/ecy.2622 (PMC6850360; doi:10.1002/ecy.2622)
Supplement: Supplementary file 2 [file ECY-100-na-s002.pdf]

**Supporting Information.** Otso Ovaskainen, Danielle Leal Ramos, Eleanor M. Slade, Thomas Merckx, Gleb Tikhonov, Juho Pennanen, Marco Aurélio Pizo, Milton Cezar Ribeiro, and Juan Manuel Morales. 2019. Joint species movement modeling: how do traits influence movements? *Ecology.*

**Appendix S2. Tests with simulated data**

***Testing the validity of the posterior sampling scheme***

To test the validity of the posterior sampling scheme, we applied it to datasets that were similar in structure to the bird data, but generated with known parameter values. In these data, we assumed that there are $n$ species, for each of which we simulated $m$ movement steps. We assumed that the species belonged to two feeding types (frugivores vs. insectivores), and that they showed variation in their body size. We randomised the log-transformed body sizes from uniform distribution in the range [2, 6], and assigned each species with equal probability to frugivores or insectivores. We first show more detailed results on a single simulated dataset, and then assess the generality of the findings by showing similar results over replicated datasets, including non-linear effects of traits on movement parameters.

*Single dataset*

We constructed a $n \times 3$ trait matrix $\mathbf{T}$ by setting the first column (i.e., values of $t_{sk}$ with $k = 1$) to intercept (all values set to one), the second column (i.e., values of $t_{sk}$ with $k = 2$) to an indicator variable describing if the species is a frugivore (with value one) or not (with value zero), and the third column (i.e., values of $t_{sk}$ with $k = 3$) to log-transformed body size. We generated a phylogenetic correlation matrix by grouping the $n$ species into $n/2$ groups, each of which contained two species. We assumed that the phylogenetic correlation within a group is 0.9, whereas the phylogenetic correlation is zero for species that do not belong to the same group.

As true parameter, we set the value of the matrix $\mathbf{Z}$ (with elements $\zeta_{kp}$) to

$$\mathbf{Z} = \begin{pmatrix} -2 & 0 & 0 \\ 1 & 0 & 2 \\ 0.5 & 0.5 & 0 \end{pmatrix}. \quad \text{(Eq. S1)}$$

The columns of the matrix $\mathbf{Z}$ correspond to the movement parameters: movement distance ($p = 1$), affinity to semi-open habitats ($p = 2$), and affinity to forests ($p = 3$). The rows of the matrix $\mathbf{Z}$ correspond to the species traits: intercept modeling the overall mean ($k = 1$), an indicator variable for frugivorous species ($k = 2$), and log-transformed body size ($k = 3$). Thus, as illustrated by Fig. S1, we assumed that movement distance is greater for frugivores than insectivores ($\zeta_{21} = 1$) and that it increases with body size ($\zeta_{31} = 0.5$), that semi-open habitats affinity increases with body size ($\zeta_{32} = 0.5$) but is not influenced by feeding type ($\zeta_{22} = 0$), and that forest affinity is greater for frugivores than insectivores ($\zeta_{23} = 2$) but it is not influenced by body size ($\zeta_{33} = 0$). We set the matrix $\mathbf{\Sigma}$ to diagonal matrix with diagonal values 0.2, 1 and 0.5. Thus, we assumed the smallest amount of variation (not explained by traits) in the parameter measuring movement distance, the largest for semi-open habitats affinity, and the second largest amount to forest affinity. We set the strength of the phylogenetic signal to $\rho = 0.7$. The realised parameter values of the species are shown at the lower row of panels in Fig. S1.

We generated simulated movement data for each species using the landscape of the bird empirical study, randomising the initial condition with equal probability among all cells, simulating $m + 10$ movement steps as a single track, and ignoring the first 10 positions. Fig. S2 illustrates such tracks for species 1-6.

We generated a large dataset (called the full data) by setting $n = 100$ and $m = 100$. We then subsampled the full data in three ways to form datasets with ($n = 100, m = 10$), ($n = 10, m = 100$), and ($n = 10, m = 10$). We fitted the model to each of the four datasets, and compared the posterior distributions of the model parameters to the true values. To examine the potential utility of using shared information across the species, we fitted the JSMM to the data in three ways, called (i) the full model, (ii) the single-

species models, and (iii) the leave-one-out models. In the full model, we fitted the model to data on all species simultaneously. In the single-species models, we fitted the model separately for each focal species, and thus included in the model parameterization data for the focal species only. In the leave-one-out models, we also parameterized the models separately for each focal species, but included in the parameterization movement data for all but the focal species. Thus, in the case of the full model, the parameter estimates for each focal species are based both on the movement data for the focal species and the information that can be borrowed from the other species. In the case of the single-species models, the parameter estimates are based solely on the movement data for the focal species. In the case of the leave-one-out models, the parameter estimates are based solely on the information that can be borrowed from the other species.

Figs. S3-S6 compare posterior distributions to the true values for the community-level parameters $\zeta$ (Fig. S3), $\mathbf{\Sigma}$ (Fig. S4), $\rho$ (Fig. S5), as well as the species-level parameters $\boldsymbol{\theta}$ (Fig. S6). In all cases, the posterior distributions become increasingly concentrated around the true values with increasing amount of data. Further, as expected, the accurate estimation of the community-level parameters requires data on many species, whereas the accurate estimation of species-specific parameters requires many movement steps for the focal species. To see this, let us first make the trivial observation that for all parameters the most accurate estimates are achieved for the case with most data, i.e. $n = 100$ and $m = 100$. For the community-level parameters (Figs. S3-S5), the second most accurate parameter estimates are obtained for $n = 100, m = 10$, and thus reducing the number of data points per species is less harmful than reducing the number of species. In contrast, for the species-level parameters (Fig. S6), the second most accurate parameter estimates are obtained for $n = 10, m = 100$, and thus reducing the number of species is less harmful than reducing the number of data points per species, naturally assuming that the species of interest are still included in the data. A comparison between the single-species models, leave-one-out models and full model in Fig. S6 shows that the benefit of borrowing information from other species can strongly depend on the parameter in question. In particular, while with these simulated data the estimation of the movement length required species specific data (single species models performed better than leave-one-out models), borrowing information from other species greatly improved the estimation of the habitat affinity parameters (leave-one-out models performed better than single species models). In all cases, the full JSMM model lead to the most accurate information, which is expected, as it can both utilize the specific data for the focal species, and additionally borrow information from the other species.

*Replicated datasets*

To test the generality of the above results, we generated 15 replicated datasets, for each of which we randomized independently the body sizes and feeding classes of the bird species, as well as the species-specific movement parameters and the actual movement data. Like above, we examined the influence of data size by subsampling each replicate data set to present either the full data ($n = 100, m = 100$), data with few movement steps per species ($n = 100, m = 10$), data with few species ($n = 10, m = 100$), or data with few movement steps for few species ($n = 10, m = 10$). As we considered 15 replicates of four sizes of data, the total number of datasets was 60. To avoid reporting an excessive number of results, we consider here only the full JSMM model, and focus on a single movement parameter, namely the movement distance, and a single trait, namely the body size. We first asked how well the fitted models were able to identify the parameter $\zeta_{31}$, i.e. the rate at which movement distance increases with body size, the true value of which is $\zeta_{31} = 0.5$. As shown by Fig. S7, this parameter can be accurately estimated for datasets that include many species ($n = 100$) but not for datasets that include only few species ($n = 10$), whereas the number of movement steps ($m = 10$ or $m = 100$) has only a minor influence. These results are consistent with those presented above for a single dataset: estimation of community level parameters requires data for many species. In the same line, the accurate estimation of the phylogenetic signal parameter is successful for cases where the data includes many species (Fig. S8).

We then asked how well the fitted models were able to identify the species-specific movement distances $\theta_{s1}$ estimated for each species $s$. As a measure of accuracy, we computed the squared error between the

true value of $\theta_{s1}$ (i.e., the one we used to simulate the data for species $s$) and its posterior mean estimate $E[\theta_{s1}]$. To obtain a single summary of estimation accuracy over the dataset, we averaged the squared errors over the species. We note that we thus ask how close the point estimates of posterior means are to the true values rather than asking e.g. how large are the credible intervals (which question we addressed in Fig. S6). As shown by Fig. S9, the identification of species-specific parameters is successful if sufficient amount of movement steps are available for each species ($m = 100$), independently of whether the dataset contains a small ($n = 10$) or a large ($n = 100$) number of species. However, in cases where only a small number of movement steps ($m = 10$) per species are available, the number of species does matter: the species-specific movement parameters are more accurate if data on many species are available ($n = 100$) compared to the case where data on only few species are available ($n = 10$). Also these results are consistent with those reported above for a single dataset: estimation of species-level parameters requires much data for each species, but having only limited amount of data per species can be partially compensated by having data for many species. This illustrates how the hierarchical structure of the model allows for "borrowing information" from the other species.

*Non-linear effects of traits on movement parameters*

To test the ability of the framework to identify non-linear effects of the traits on movement parameters, we added squared body size as a fourth trait ($t = 4$), and considered two alternative matrices $\mathbf{Z}$, denoted by $\mathbf{Z}^{\mathrm{L}}$ (linear case) and $\mathbf{Z}^{\mathrm{NL}}$ (non-linear case). For the linear case, we defined

$$\mathbf{Z}^{\mathrm{L}} = \begin{pmatrix} -2 & 0 & 0 \\ 1 & 0 & 2 \\ 0.5 & 0.5 & 0 \\ 0 & 0 & 0 \end{pmatrix}. \qquad \text{(Eq. S2)}$$

As we assumed that squared body size does not influence any of the movement parameters (the fourth row of $\mathbf{Z}^{\mathrm{L}}$ consists of zeros), and as the matrix of Eq. S2 is otherwise identical to that of Eq. S1, the parameters of the linear case are identical to those of applied above.

For the non-linear case, we defined

$$\mathbf{Z}^{\mathrm{NL}} = \begin{pmatrix} -7 & 0 & 0 \\ 1 & 0 & 2 \\ 4 & 0.5 & 0 \\ -0.5 & 0 & 0 \end{pmatrix}, \qquad \text{(Eq. S3)}$$

and thus assumed otherwise identical links from traits to movement parameters (Fig. S1), except that the movement distance is now assumed to behave non-linearly with body size, being largest for birds with intermediate body size (Fig. S10). As above, we considered constructed four sizes of subsampled data for 15 replicates of both the linear and non-linear cases, the total number of datasets being now 120 (out of which the 60 datasets representing the linear case are identical to those analysed before).

Here our aim was to ask whether a researcher could identify the non-linear relationship between body size and movement distance, and thus we focus on the parameter $\zeta_{41}$, the true value of which is $\zeta_{41} = 0$ for the linear case and $\zeta_{41} = -0.5$ for the non-linear case. Fig. S11 shows that the identification of the non-linear effect was successful for datasets that included many species ($n = 100$) whereas it was not successful for datasets that included few species ($n = 10$), the effect of the movement steps per species ($m = 10$ or $m = 100$) having a milder influence. Thus, these results are in line with those presented above: identifying community-level parameters (such as the presence of a non-linear relationship between traits and movement parameters) requires data on many species. When analysing real data, a researcher hypothesizing a non-linear relationship between species traits and movement parameters could add the second order of body size as a trait, as we did here with simulated data. Depending on whether or not the fitted model gives support for such a non-linear relationships, the researcher may then decide to either keep the non-linear relationship in the final model or to leave it out from the final model.

***Examining model assumptions with posterior predictive checking***

The JSMM framework is flexible in the sense that one can combine the community-level model (Eq. 1 in the main paper) with almost any kind of a movement model. For example, we fitted the bird data to a stochastic redistribution model, and the moth data to a spatially structured diffusion model. These models, like any movement models, make many kinds of structural assumptions, the validity of which are typically not known *a priori*. If the structural model assumptions are seriously violated, the inference derived by fitting the model to the data may be invalid, and thus the validity of the assumptions should be checked. We note that this consideration is not specific to JSMM, but for any modelling approach. For example, in case of the linear model, the inference (say, p-values) may not be valid unless the underlying assumptions hold, namely those of linearity, homoscedasticity of residuals, and independence of the data points. With more complex models, such as the movement models considered here, there is no pre-defined list of assumptions one needs to check and especially how to do that. As one general method for checking structural model assumptions, we illustrate here posterior predictive checking for the bird case study.

The general idea of posterior predictive checking is to generate simulated datasets that have the same structure as the real dataset, and then ask whether the real dataset somehow systematically differs from the simulated datasets. To do so, we first sampled model parameters (i.e., the vector of all model parameters, including both community- and species-level parameters) from their joint posterior distribution. We then "released" each bird individual to the location where it was first observed in the real data, and simulated the same number of movement steps for each individual as observed in the real data. We repeated this procedure 100 times, always sampling a new parameter combination from the posterior distribution. In this way, the 100 simulated data sets include both parameter uncertainty (we sampled the parameters repeatedly from the posterior) as well as process uncertainty (we randomized the next location of the bird based on the movement probabilities, thus resulting in different realizations even with identical parameters). However, the simulated datasets assume a certain model structure (Eq. 2 in the main paper), for which reason the simulated datasets may differ systematically from the real one, even if they are based on a model fitted to the data.

As one example, let us consider the assumption that the lengths of the movement steps are exponentially distributed, i.e. the term $\exp(-d_{ij}/\alpha_s)$ in Eq. 2 of the main paper. Equally well, we could have assumed a Gamma, Weibull, Gaussian, or some other distribution. The question to ask is not which theoretical distribution is the correct one, as the birds are unlikely to follow exactly any pre-defined statistical distribution, but whether e.g. the exponential distribution is "good enough" to replicate the main features of the data. For example, if the birds would in reality take half of the times steps of 10 meters, and half of the time steps of 1000 meters, the exponential distribution would clearly not be adequate. To examine if the exponential distribution is a "good enough" approximation, we can select some summary statistics of the data that would be informative about the distribution of movement lengths. For example, we may consider separately for each species the distribution of all movement step lengths, and compute their mean, minimum, and maximum. Computing these over the 100 simulated datasets results in posterior predictive distributions of the variables of interest, which we may compare to the observed values. As one example, in the observed data the maximum distance moved by *Empidonomus varius* is 60 meters, whereas in the posterior predictive distribution the mean of this variable is 84 meters, and the 95% credibility interval is from 40 meters to 170 meters. As the observed value fits well within the predicted distribution, this test did not yield evidence of model misspecification. Repeating the same tests for all species, and for minimum, mean and maximum distances (Figs. S12 and S13) yields the same result: the observed data is not systematically different from the simulated data in terms of the step length distributions, and thus we may consider the assumed exponential distribution to be sufficiently realistic to be able to replicate the data.

The posterior predictive test presented above is of course just one example of the many tests that one could make. We could have equally well compared the observed data to the posterior predictive data in terms of e.g. the presence of autocorrelation among consecutive movement steps: the model assumes

random walk but maybe the individuals display home-range behaviour. Or we could have examined variability among individuals within species: for many species we have data on several individuals, which we assumed to behave identically, but maybe there is substantial variation among individual in their movement characteristics. As a third example, we could have examined variability in movement characteristics over time: in the model we ignored e.g. variation in weather conditions, which may have a strong influence on movements. While more comprehensive validation of structural model assumptions should be done in specific case studies, we illustrated here only the general principle of posterior predictive checking as the aim of the present paper was to present the overall JSMM framework.

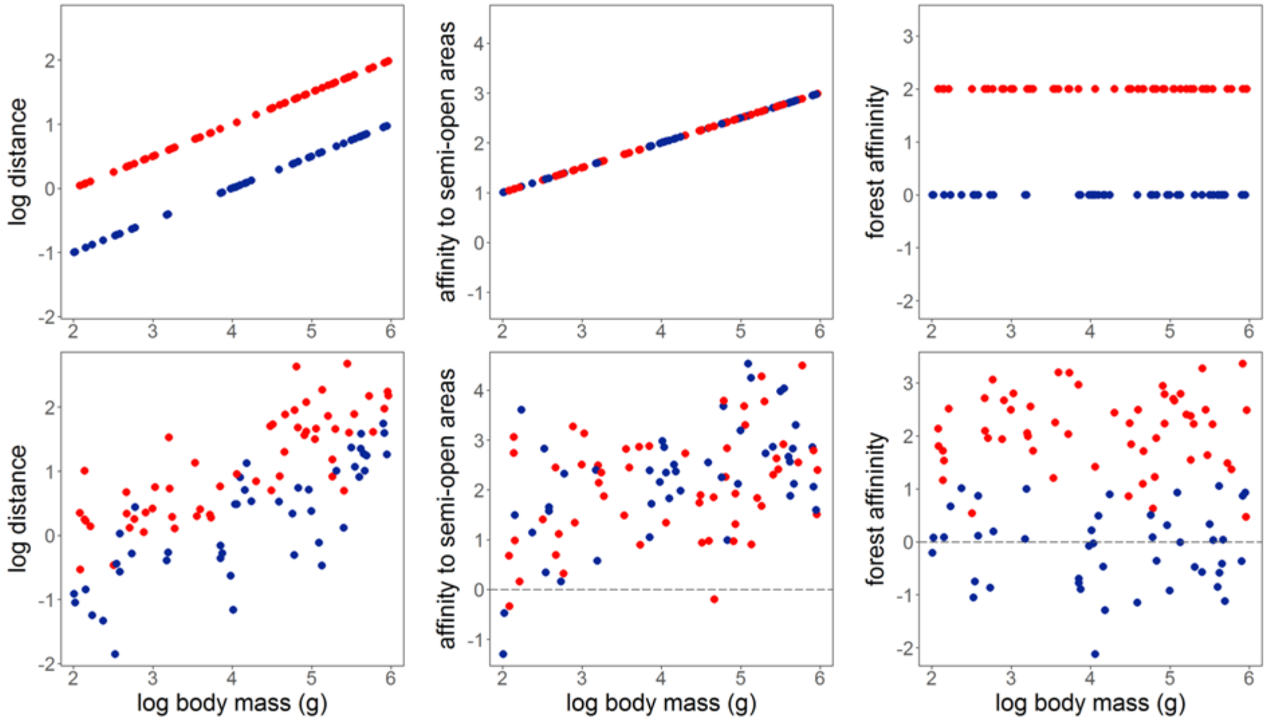


**Figure S1.** Species-specific movement parameters and their relationship to species traits assumed in the simulated case study. In each panel, one dot corresponds to one species. The upper row of panels shows the expected movement parameters based on the species traits, and the lower row of panels the realised movement parameters, based on both traits and the residual variation that is structured by phylogenetic relationships. Frugivores are depicted by red and insectivores by blue colour.

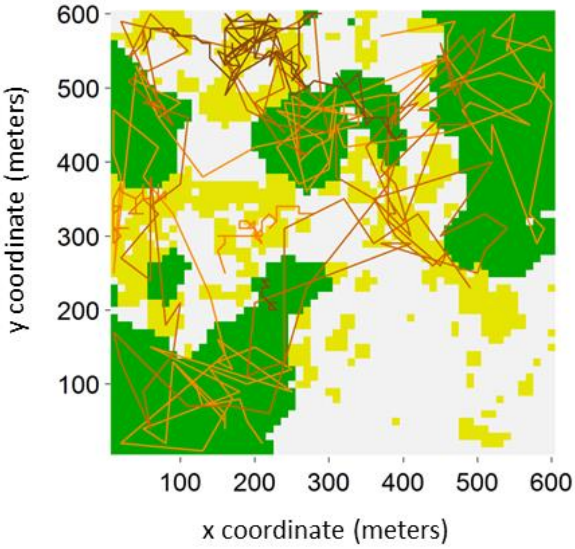


**Figure S2.** Illustration of simulated movement tracks. Each line shows $m = 100$ simulated movement steps. Out of the full community with $n = 100$ species, the data are shown for species 1-6, each species being shown by one colour.

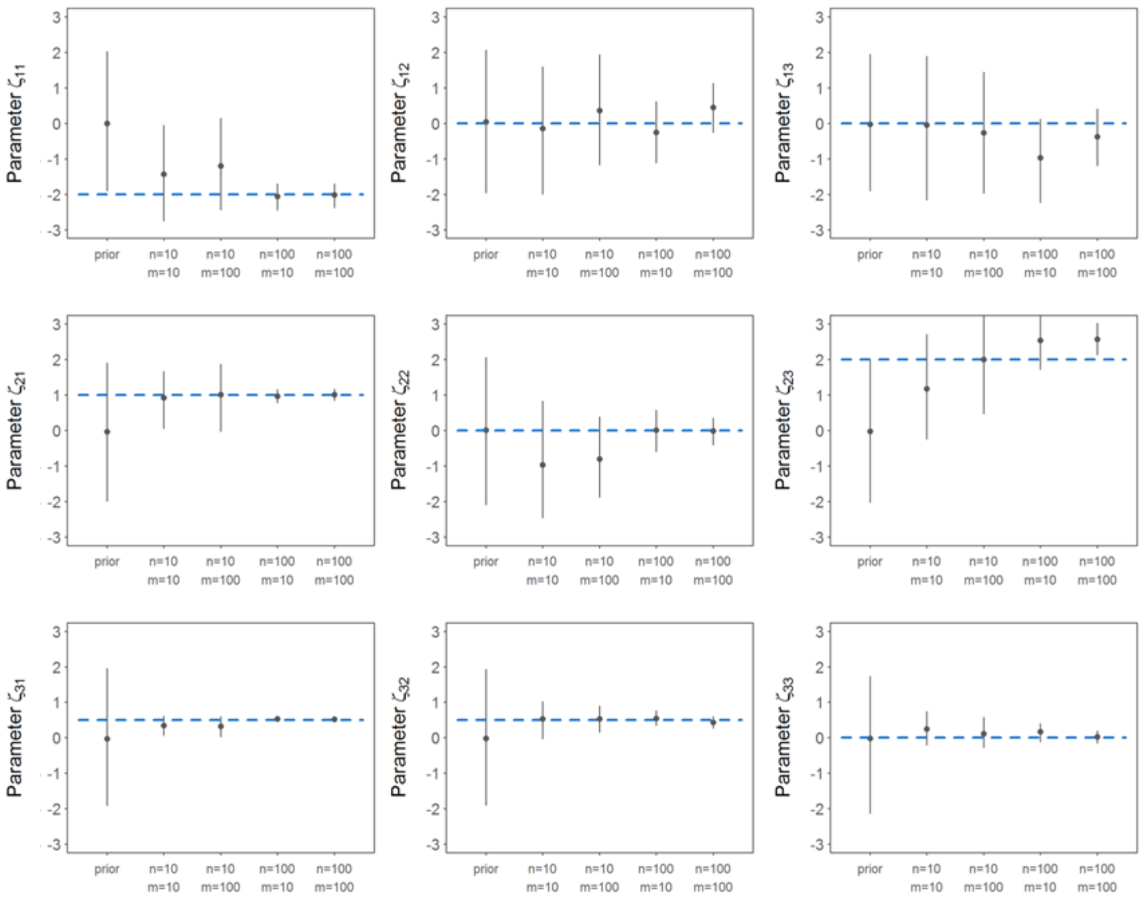


**Figure S3.** The ability of the model to identify the true values of the parameters $\zeta$ measuring the influence of species traits on movement parameters. In each panel, the dashed line shows the true value, and the dots and the lines show the mean and 95% quantile of the prior and posterior distributions. The posterior distributions are shown for four scenarios for data availability, with either $n = 10$ or $n = 100$ species, and with either m= $10$ or $m = 100$ movements steps per species.

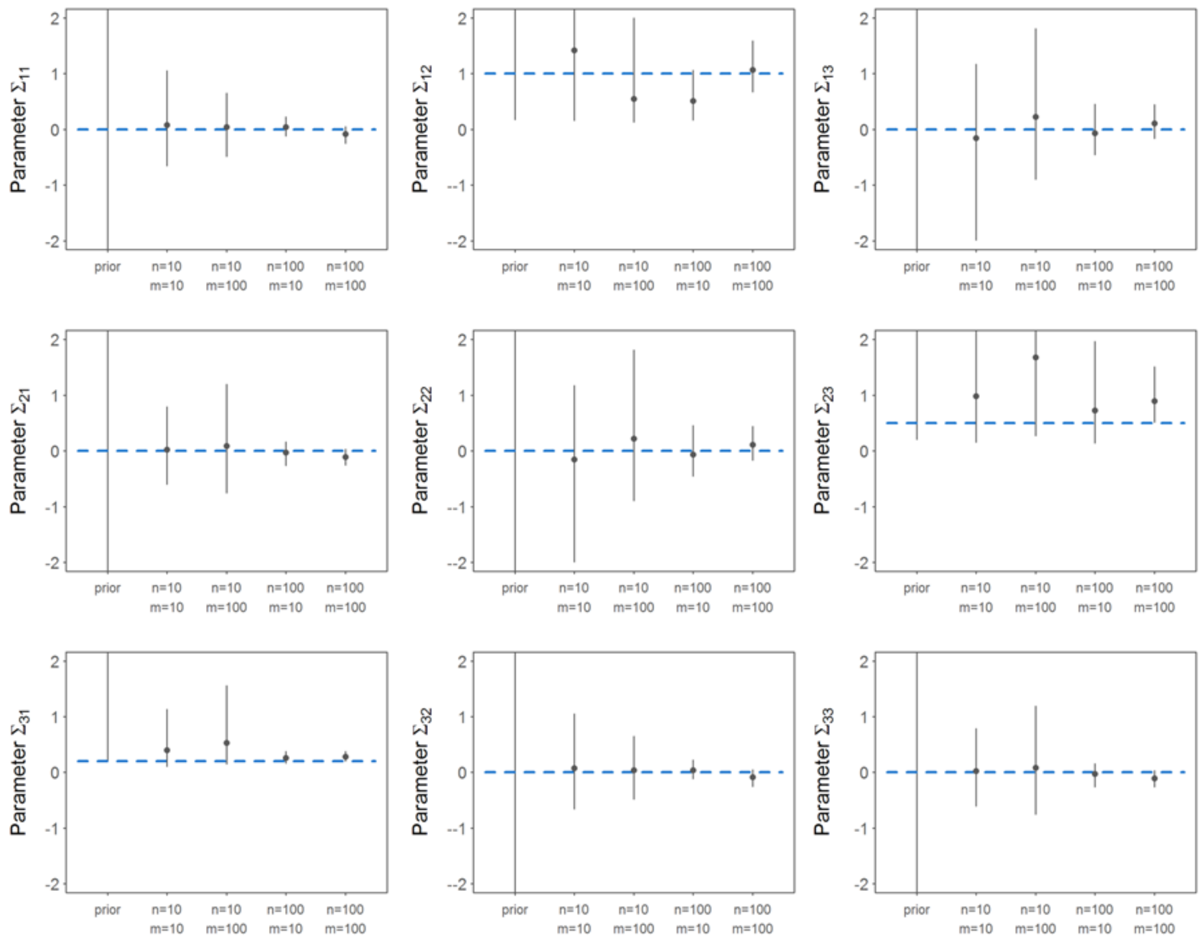


**Figure S4.** The ability of the model to identify the true value of the parameter $\mathbf{\Sigma}$ measuring the amount of variation in movement parameters among the species not explained by traits. In each panel, the dashed line shows the true value, and the dots and the lines show the mean and 95% quantile of the prior and posterior distributions. The posterior distributions are shown for four scenarios for data availability, with either $n = 10$ or $n = 100$ species, and with either m$= 10$ or $m = 100$ movements steps per species.

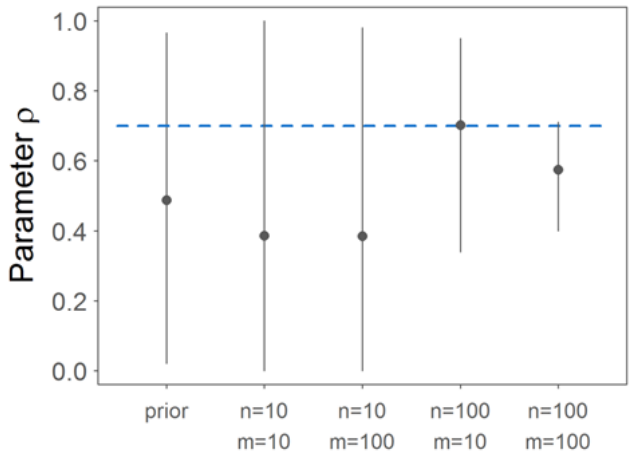


**Figure S5.** The ability of the model to identify the true value of the parameter $\rho$ measuring the strength of phylogenetic signal in variation of the movement parameters among the species. The dashed line shows the true value, and the dots and the lines show the mean and 95% quantile of the prior and posterior distributions. The posterior distributions are shown for four scenarios for data availability, with either $n = 10$ or $n = 100$ species, and with either m$= 10$ or $m = 100$ movements steps per species.

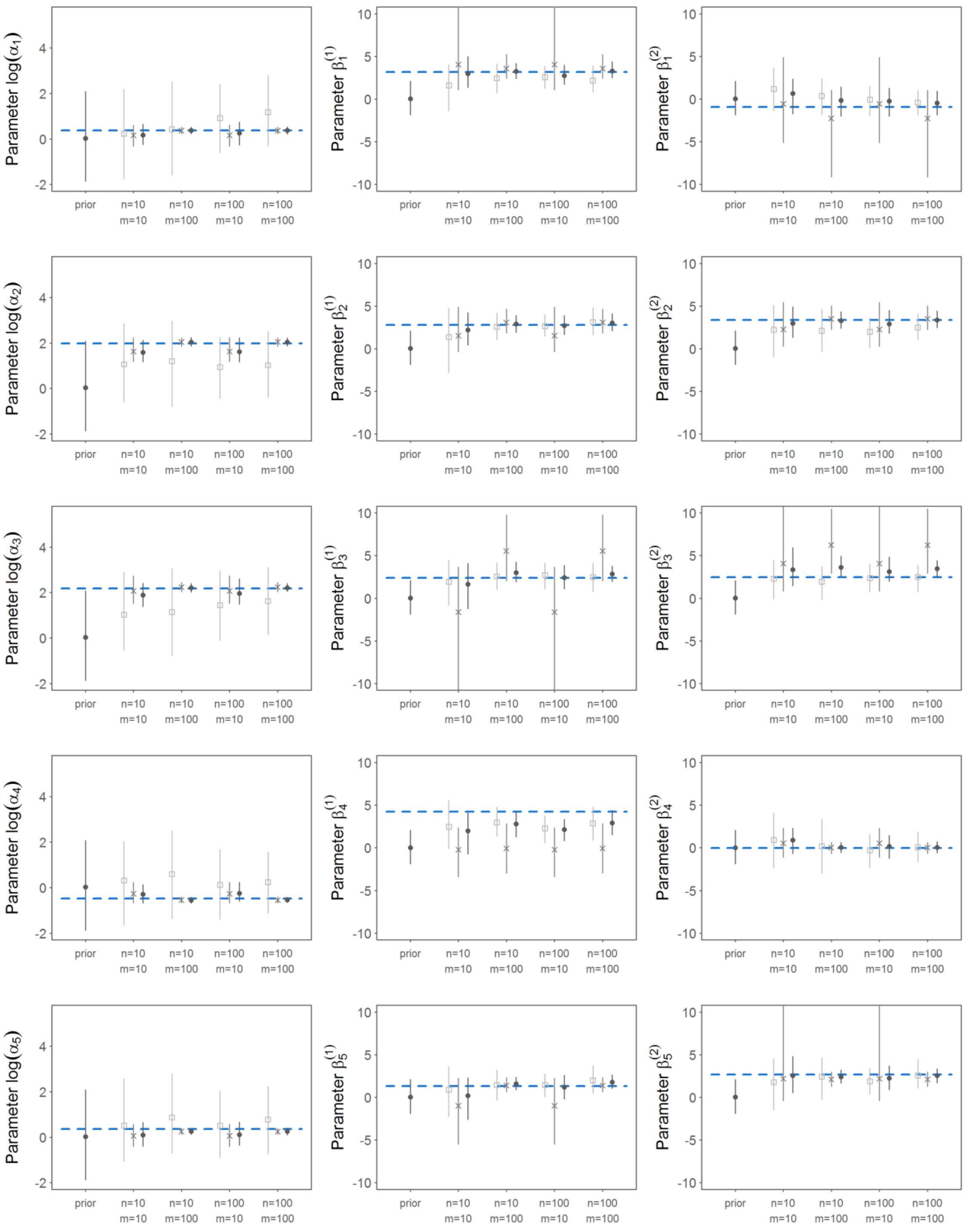


**Figure S6.** The ability of the model to identify the true values of the species-level movement parameters $\boldsymbol{\theta}$. In each panel, the dashed line shows the true value, and the dots and the lines show the mean and 95% quantile of the prior and posterior distributions. The posterior distributions are shown for four scenarios for data availability, with either $n = 10$ or $n = 100$ species, and with either m= $10$ or $m = 100$ movements steps per species. The bars (from left to right) show the results for the leave-one out models (light grey bar with circle), for single species models (grey bar with cross), and for the full model (black bar and dot). The parameter estimates are shown for five species included in all data sets.

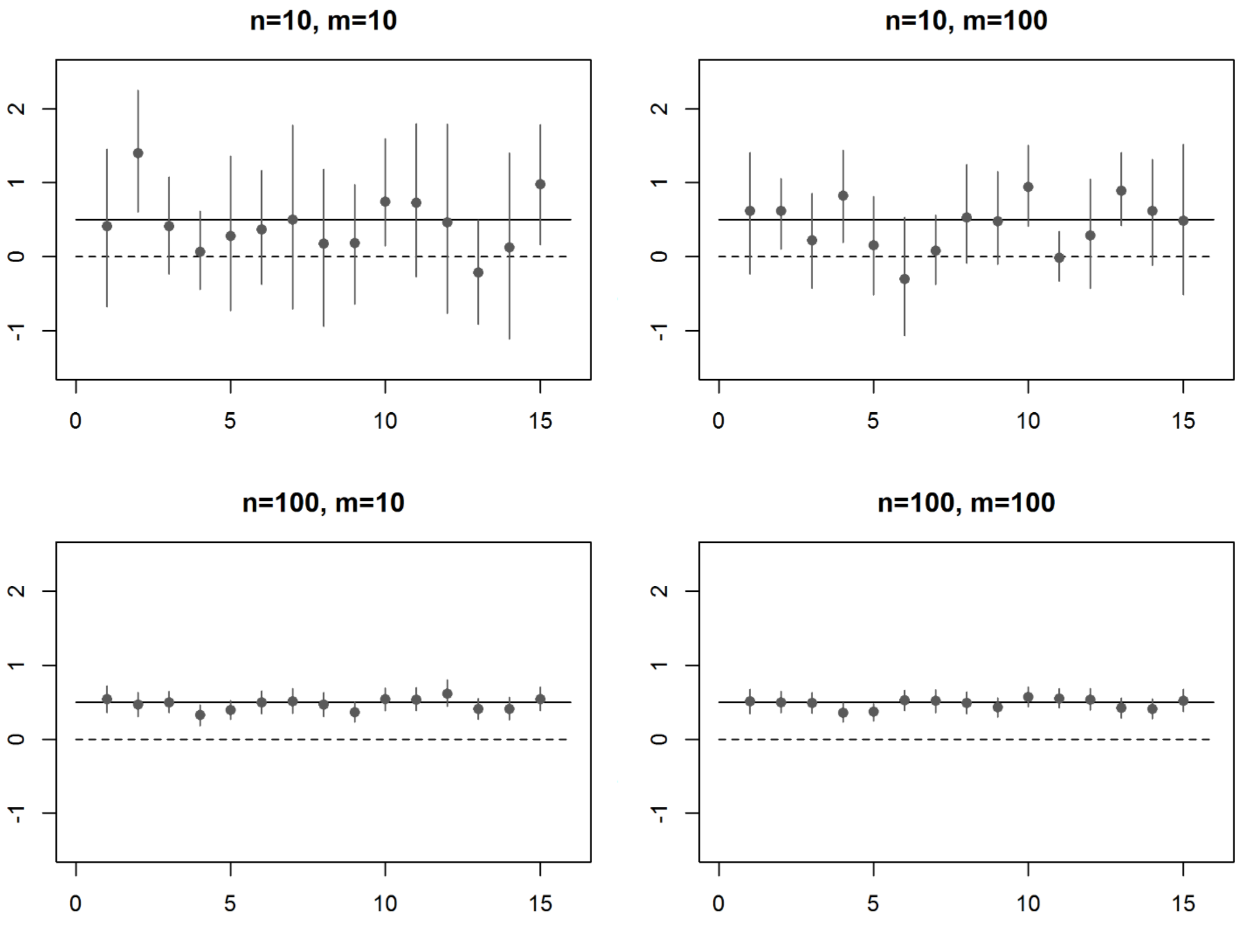


**Figure S7.** The ability of the JSMM framework to identify the influence of species traits on movement parameters. Shown are posterior distributions (dot corresponding to the mean and the line to the 95% credibility interval) for the parameter $\zeta_{31}$ that measures the influence of body size to movement distance (see text). Each panel shows the results for 15 replicates of the data, the solid line showing the true value ($\zeta_{31} = 0.5$) and the dashed line showing zero.

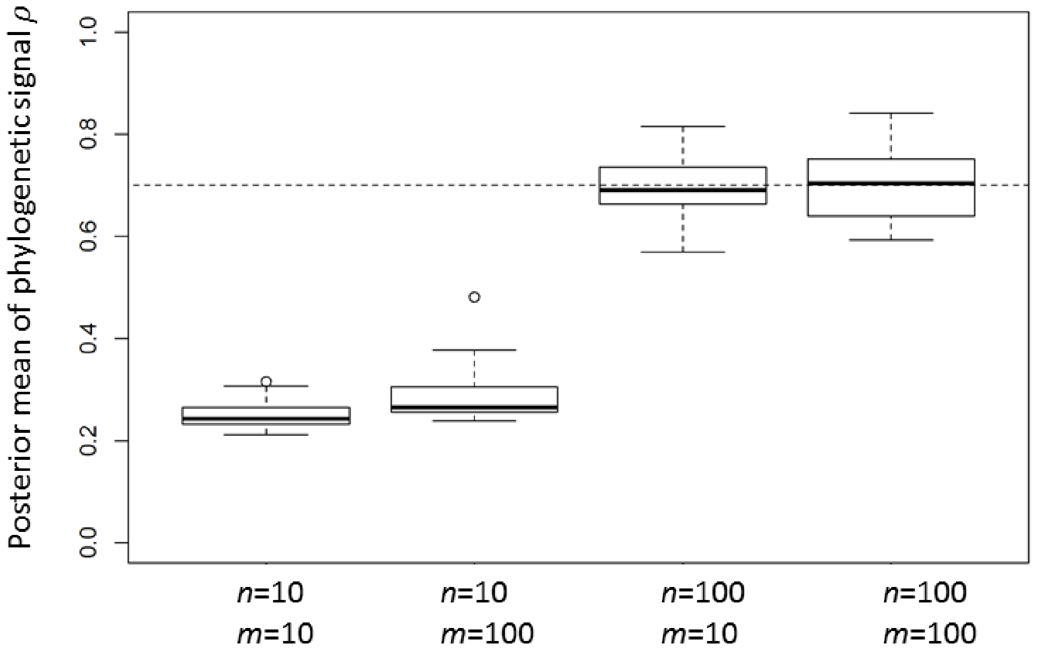


**Figure S8.** The ability of the JSMM framework to identify the strength of phylogenetic signal in the data. Shown is the posterior mean estimate of the parameter $\rho$. Each box-plot shows the result for 15 replicates of the data set, with either $n = 10$ or $n = 100$ species, and with either m$= 10$ or $m = 100$ movements steps per species. The dashed line shows the true value ($\rho = 0.7$) assumed when generating the simulated data.

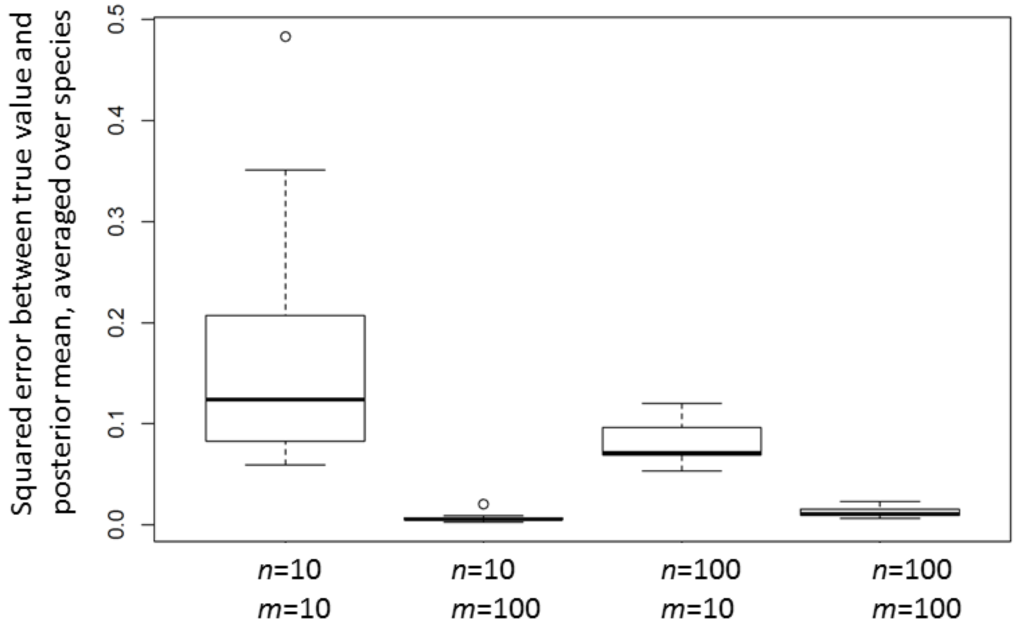


**Figure S9.** The ability of the JSMM framework to identify species-specific movement parameters. Shown is the average (over the $n$ species) squared difference between the true value of the parameter $\theta_{s1}$ and its posterior mean. As described in the text, this parameter models the movement distance for species $s$. Each box-plot shows the result for 15 replicates of the data set, with either $n = 10$ or $n = 100$ species, and with either m$= 10$ or $m = 100$ movement steps per species.

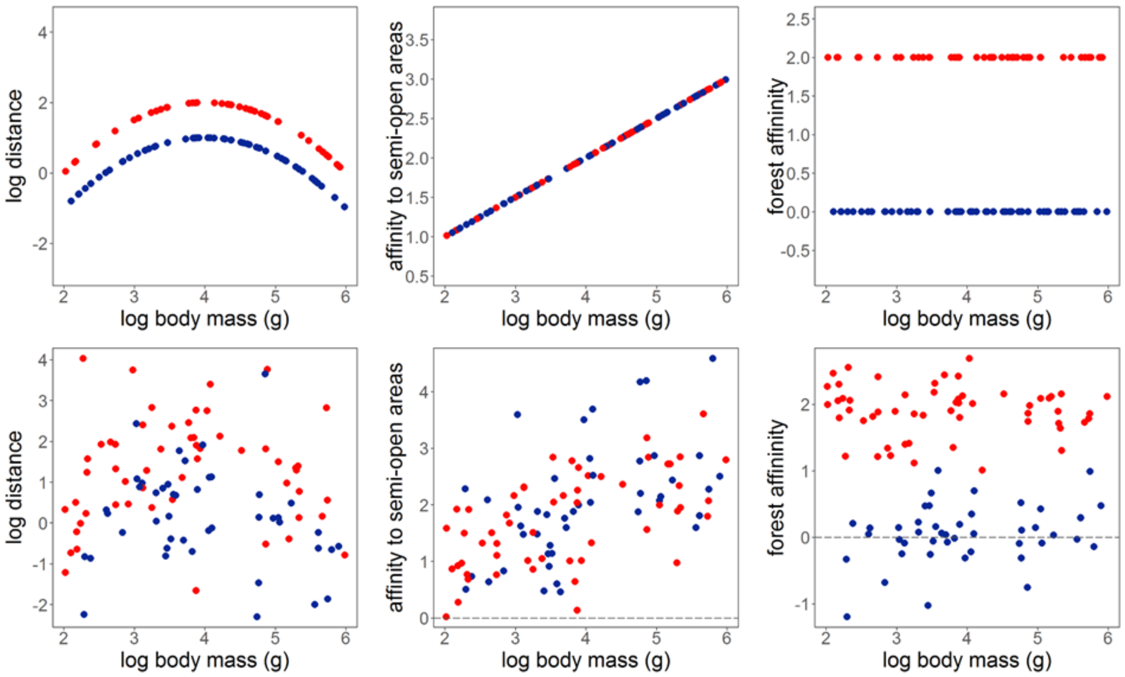


**Figure S10.** Species-specific movement parameters and their relationship to species traits assumed in the simulated case study with non-linear effects of body size on movement distance. In each panel, one dot corresponds to one species. The upper row of panels shows the expected movement parameters based on the species traits, and the lower row of panels the realised movement parameters, based on both traits and the residual variation that is structured by phylogenetic relationships. Frugivores are depicted by red and insectivores by blue colour.

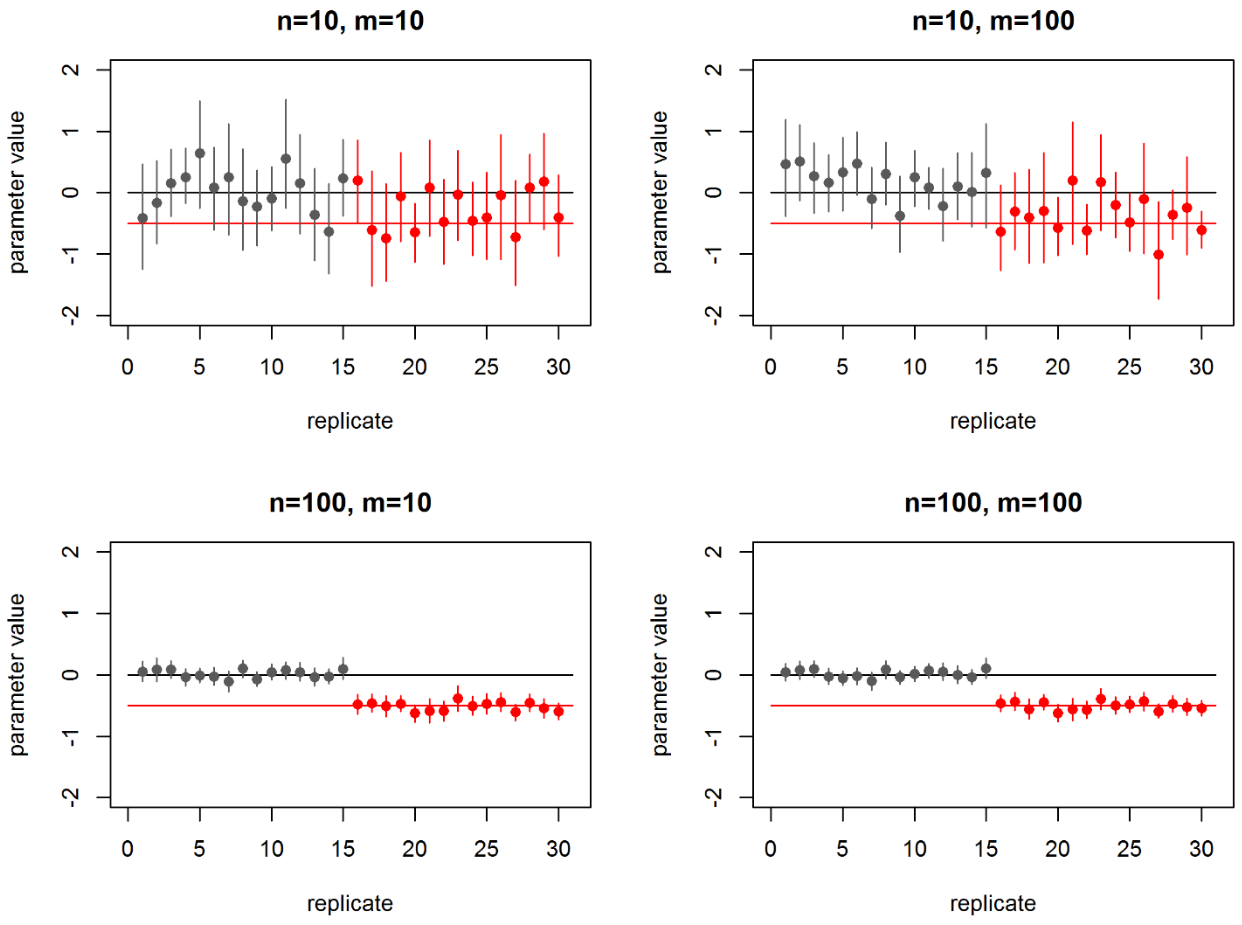


**Figure S11.** The ability of the JSMM framework to identify non-linear relationship between species traits and movement parameters. Shown are posterior distributions (dot corresponding to the mean and the line to the 95% credibility interval) for the parameter $\zeta_{41}$ that measures the influence of squared body size to movement distance (see text). Each panel shows the results for 15 replicates of the data generated by the linear model (black colour, $\zeta_{41} = 0$) and for 15 replicates of the data generated by the non-linear model (red colour, $\zeta_{41} = -0.5$).

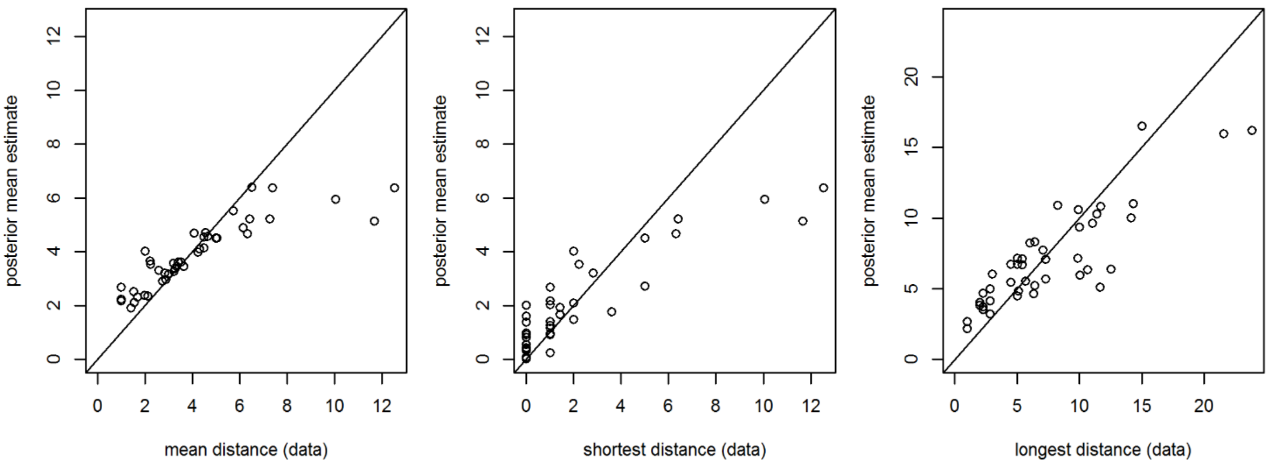


**Figure S12.** An example of posterior predictive checks for the bird case study. For each species, we computed the distribution of all observed movement distances, of which we took the mean distance (left panel), the shortest distance (middle panel), and the longest distance (right panel). We then computed the mean, shortest and longest distances for 100 replicate datasets, each of which was simulated with movement parameters sampled from the posterior distribution. The simulated datasets mimicked the real datasets as close as possible in the sense that we “released” each individual to where it was first observed, and we simulated the same number of movement steps for each individual as observed in the real data. In the panels each dot corresponds to one species, and the panels compare the observed values of the (mean, minimum or maximum) movement distances (x-axis) to the posterior means estimates. The lines show the identity line y=x.

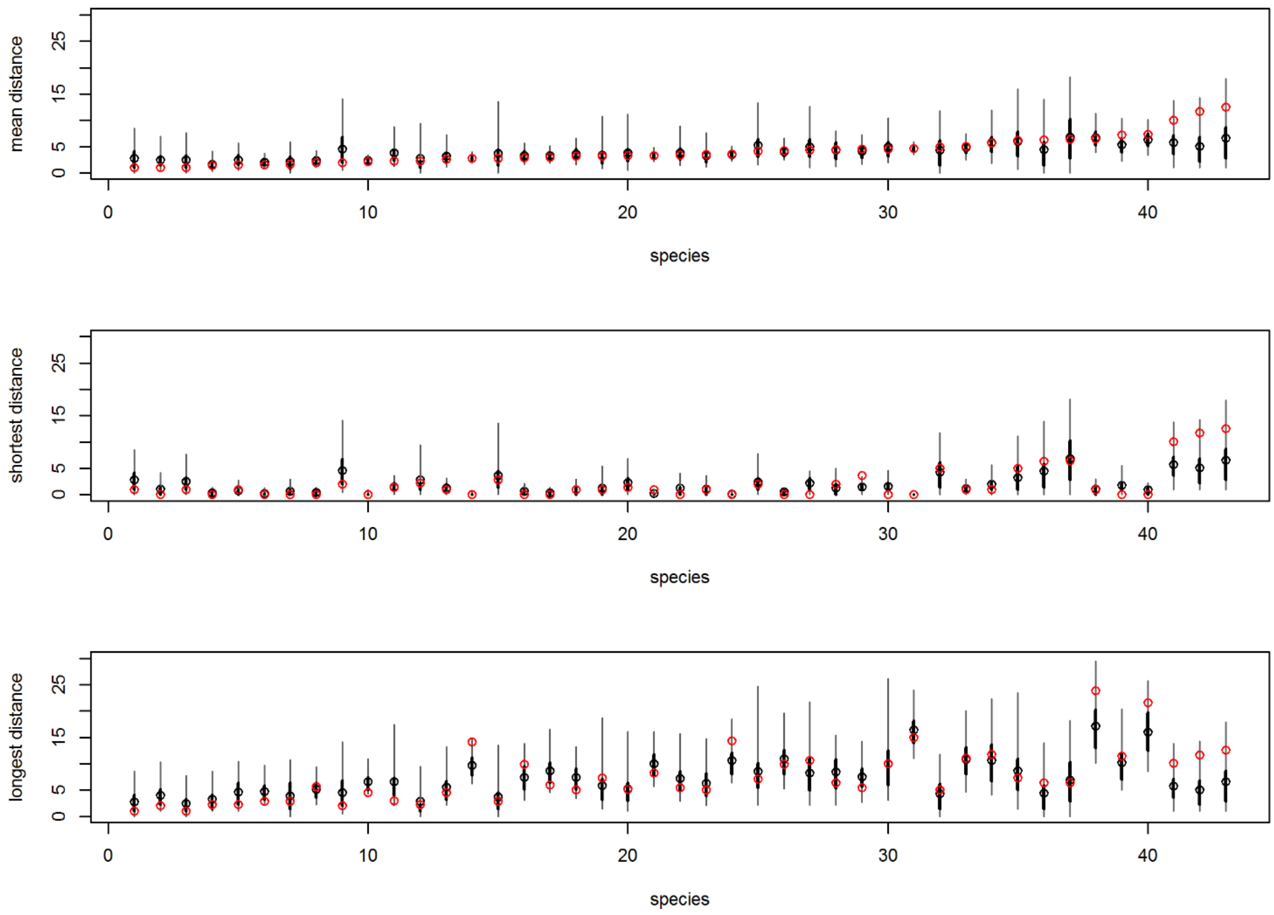


**Figure S13.** An example of posterior predictive checks for the bird case study. The figure illustrates on the same data described in Fig. S12, but now the full posterior distribution (black dots show the mean, black line the [0.25,0.75] quantile and grey line the [0.025,0.975] quantile) of the (mean, minimum or maximum) movement distance is shown for each species. The red dots show the observed values, and the species are ordered according to the observed mean distance. The true value is located within the [0.25,0.75] quantile (including the endpoints) for 84% (mean distance), 79% (shortest distance) and 60% (longest distance) of the species, and it is located within the [0.025,0.975] quantile (including the endpoints) for 100% (mean distance), 98% (shortest distance) and 100% (longest distance) of the species.
