# Supplementary material for: Joint species movement modeling: how do traits influence movements?
Source: Ecology. 2019 Feb 21;100(4):e02622. doi: 10.1002/ecy.2622 (PMC6850360; doi:10.1002/ecy.2622)
Supplement: Supplementary file 3 [file ECY-100-na-s003.pdf]

Supporting Information. Otso Ovaskainen, Danielle Leal Ramos, Eleanor M. Slade, Thomas Merckx, Gleb Tikhonov, Juho Pennanen, Marco Aurélio Pizo, Milton Cezar Ribeiro, and Juan Manuel Morales. 2019. Joint species movement modeling: how do traits influence movements? *Ecology.*

Appendix S3. Detailed information on the case studies.

Tables S1 and S2 list the species and their traits, as well as the amount of data per species for the bird (Table S1) and moth (Table S2) case studies.

To estimate the parameters, we ran a total of 100,000 iterations, with a 20,000 burn-in period and thinning interval of 10. We ran three chains and checked the convergence of the posteriors using the Gelman-Rubin convergence statistic (Gelman and Rubin, 1992), with the package CODA (Plummer et al., 2006), convergence results shown in Tables S3 and S4.

Table S1. Bird species numerical identification, name, feeding class, body size (g), and number of steps observed.

ID	Species name	Feeding class	Body size	N. steps
1	*Empidonomus varius*	Insectivorous	27.1	19
2	*Columbina talpacoti*	Granivorous	46.0	5
3	*Tangara cayana*	Frugivorous	18.0	37
4	*Tachyphonus coronatus*	Frugivorous	29.3	34
5	*Pachyramphus validus*	Insectivorous	43.0	6
6	*Cyanocorax cristatellus*	Omnivorous	178.0	5
7	*Camptostoma obsoletum*	Insectivorous	8.1	3
8	*Myiodynastes maculatus*	Insectivorous	43.2	11
9	*Euphonia chlorotica*	Frugivorous	11.0	5
10	*Mimus saturninus*	Insectivorous	63.7	1
11	*Cnemotriccus fuscatus*	Insectivorous	13.6	1
12	*Pitangus sulphuratus*	Omnivorous	62.9	7
13	*Cyclarhis gujanensis*	Insectivorous	28.8	12
14	*Dacnis cayana*	Frugivorous	13.0	27
15	*Xolmis velatus*	Insectivorous	49.1	9
16	*Vireo chivi*	Insectivorous	16.1	5
17	*Tyrannus melancholicus*	Insectivorous	37.4	7
18	*Brotogeris chiriri*	Granivorous	61.6	5
19	*Colaptes melanochloros*	Insectivorous	127.3	1
20	*Zonotrichia capensis*	Granivorous	20.3	7
21	*Megarynchus pitangua*	Insectivorous	69.9	15
22	*Piaya cayana*	Insectivorous	102.0	13
23	*Gnorimopsar chopi*	Omnivorous	65.9	1
24	*Molothrus bonariensis*	Insectivorous	41.5	6
25	*Nemosia pileata*	Insectivorous	16.0	12
26	*Euphonia violacea*	Frugivorous	15.0	1
27	*Euphonia cyanocephala*	Frugivorous	14.0	3
28	*Conirostrum speciosum*	Insectivorous	8.8	5
29	*Myiozetetes similis*	Insectivorous	28.0	1
30	*Tersina viridis*	Frugivorous	29.0	5
31	*Melanerpes candidus*	Insectivorous	108.0	1

32	*Colaptes campestris*	Insectivorous	158.0	3
33	*Myiarchus ferox*	Insectivorous	27.5	1
34	*Basileuterus flaveolus*	Insectivorous	13.2	1
35	*Forpus xanthopterygius*	Granivorous	31.0	3
36	*Saltator similis*	Omnivorous	43.3	1
37	*Nystalus chacuru*	Insectivorous	52.3	3
38	*Dryocopus lineatus*	Insectivorous	183.2	1
39	*Turdus leucomelas*	Omnivorous	69.1	65
40	*Turdus rufiventris*	Omnivorous	69.4	35
41	*Tangara sayaca*	Frugivorous	32.5	109
42	*Turdus amaurochalinus*	Omnivorous	57.9	5
43	*Patagioenas picazuro*	Granivorous	279.0	20

Table S2. Moth species numerical identification, name, habitat preference, adult diet, wing span, number of marked individuals, and total number of recaptures by species.

ID	Species name	Habitat preference	Adult diet	Wing span (mm)	N. marked individuals	Total n. of recaptures
1	*Phlogophora meticulosa*	open	feeding	50	2	0
2	*Ennomos quercinaria*	semi-open	not feeding	38	69	0
3	*Autographa pulchrina*	open	feeding	40	27	2
4	*Laspeyria flexula*	semi-open	not feeding	30	12	1
5	*Lymantria monacha*	forest	not feeding	44	418	42
6	*Timandra comae*	semi-open	not feeding	33	33	0
7	*Plemyria rubiginata*	semi-open	not feeding	28	11	0
8	*Lacanobia oleracea*	open	not feeding	38	81	3
9	*Opisthograptis luteolata*	open	not feeding	35	55	0
10	*Noctua fimbriata*	semi-open	feeding	60	20	0
11	*Rusina ferruginea*	open	feeding	38	54	3
12	*Habrosyne pyritoides*	semi-open	feeding	40	164	5
13	*Spilosoma luteum*	open	not feeding	40	104	1
14	*Eilema depressa*	semi-open	not feeding	35	49	0
15	*Phalera bucephala*	semi-open	not feeding	50	48	5
16	*Diachrysia chrysitis*	open	feeding	40	151	19
17	*Mythimna ferrago*	open	feeding	38	70	6
18	*Lomaspilis marginata*	open	not feeding	27	14	2
19	*Apamea epomidion*	semi-open	feeding	40	104	1
20	*Lomographa temerata*	semi-open	not feeding	28	10	0
21	*Apamea crenata*	open	feeding	42	7	0
22	*Hemithea aestivaria*	semi-open	feeding	30	31	0
23	*Eilema lurideola*	open	feeding	32	862	61
24	*Pharmacis lupulina*	open	not feeding	30	132	3
25	*Craniophora ligustri*	semi-open	feeding	37	189	5
26	*Apamea monoglypha*	open	feeding	50	623	10
27	*Eilema griseola*	semi-open	feeding	35	954	35
28	*Melanchra persicariae*	open	feeding	40	12	0
29	*Xestia triangulum*	semi-open	feeding	42	1022	166

30	*Euthrix potatoria*	semi-open	not feeding	55	609	37
31	*Cosmia trapezina*	semi-open	feeding	34	1316	131
32	*Selenia dentaria*	semi-open	not feeding	43	162	0
33	*Deilephila elpenor*	open	feeding	65	5	0
34	*Smerinthus ocellata*	open	not feeding	80	4	0
35	*Tethea ocularis*	semi-open	feeding	38	3	0
36	*Axylia putris*	open	feeding	34	107	5
37	*Ochropleura plecta*	open	feeding	30	226	2
38	*Arctia caja*	open	not feeding	70	3	1
39	*Phymatopus hecta*	semi-open	not feeding	32	5	0
40	*Anaplectoides prasina*	forest	feeding	48	73	0
41	*Pseudoips prasinana*	forest	not feeding	36	1	0
42	*Agrotis exclamationis*	open	feeding	38	786	41
43	*Scoliopteryx libatrix*	open	feeding	46	21	0
44	*Diarsia mendica*	forest	feeding	35	176	13
45	*Hydriomena furcata*	open	not feeding	32	894	22
46	*Malacosoma neustria*	semi-open	not feeding	32	5	0
47	*Geometra papilionaria*	semi-open	not feeding	55	4	0
48	*Apamea anceps*	open	feeding	43	16	0
49	*Noctua pronuba*	open	feeding	55	718	6
50	*Apamea lithoxylaea*	semi-open	feeding	45	11	0
51	*Campaea margaritata*	semi-open	not feeding	40	442	8
52	*Stauropus fagi*	forest	not feeding	60	74	4
53	*Cyclophora punctaria*	forest	feeding	28	18	0
54	*Colocasia coryli*	forest	not feeding	35	89	3
55	*Watsonalla binaria*	semi-open	not feeding	30	1	0
56	*Pterostoma palpina*	open	not feeding	43	69	4
57	*Calliteara pudibunda*	open	not feeding	42	3	0
58	*Thyatira batis*	semi-open	feeding	35	61	0
59	*Drepana falcataria*	semi-open	not feeding	35	1	0
60	*Biston betularia*	semi-open	not feeding	55	14	0
61	*Autographa jota*	open	feeding	40	3	0
62	*Laothoe populi*	open	not feeding	80	159	17
63	*Melanthia procellata*	semi-open	not feeding	40	46	1
64	*Sphinx ligustri*	semi-open	not feeding	110	1	0
65	*Selenia tetralunaria*	semi-open	not feeding	44	98	4
66	*Phragmatobia fuliginosa*	open	not feeding	35	16	0
67	*Apamea sordens*	open	feeding	40	29	0
68	*Crocallis elinguaria*	semi-open	not feeding	38	163	8
69	*Bena bicolorana*	forest	feeding	45	1	0
70	*Ligdia adustata*	semi-open	not feeding	25	15	0
71	*Plagodis dolabraria*	semi-open	feeding	35	2	0
72	*Xestia c-nigrum*	open	feeding	36	184	1
73	*Hada plebeja*	semi-open	feeding	35	4	1
74	*Agrotis puta*	open	feeding	30	5	0
75	*Autographa gamma*	open	feeding	40	16	0

76	*Apamea scolopacina*	forest	feeding	35	127	4
77	*Euplexia lucipara*	open	feeding	33	20	0
78	*Hemistola chrysoprasaria*	semi-open	not feeding	35	2	0
79	*Herminia grisealis*	semi-open	feeding	25	29	0
80	*Abrostola tripartita*	open	feeding	33	19	0
81	*Ourapteryx sambucaria*	semi-open	not feeding	54	46	1
82	*Charanyca trigrammica*	open	not feeding	35	22	1
83	*Spilosoma lubricipeda*	open	not feeding	40	38	0
84	*Leucoma salicis*	semi-open	not feeding	45	1	0
85	*Lomographa bimaculata*	semi-open	not feeding	28	3	0
86	*Camptogramma bilineata*	open	feeding	27	2	0
87	*Euproctis similis*	open	not feeding	33	722	24

Table S3. Parameters estimates and convergence diagnostics of the MCMC approach used to sample the posterior distribution for the birds case study with real data. The estimated parameters are ρ, $\mathbf{\Sigma}$, ζ_{kp}, α_s, $\beta_s^{(1)}$ and $\beta_s^{(2)}$, where $k = 1, \dots, 5$ represents the trait, $s = 1, \dots, 43$ the species, and $p = 1,2,3$ the movement parameter. We show for each parameter the posterior mean, the 0.025 and 0.975 quantiles, the potential scale reduction factor (Rhat), the upper 95% confidence limit (95% C.I.) and the effective sample size (n.eff) for each parameter.

Parameter	Mean	0.025 quantile	0.975 quantile	Rhat	95% C.I.	n.eff
ρ	0.22	0.00	0.87	1.00	1.01	1005.5
Σ_{11}	0.20	0.09	0.37	1.00	1.01	3000.0
Σ_{12}	0.02	-0.70	0.74	1.02	1.04	784.7
Σ_{13}	-0.12	-0.87	0.52	1.02	1.05	947.4
Σ_{21}	0.02	-0.70	0.74	1.02	1.04	784.7
Σ_{22}	7.47	0.55	23.92	1.02	1.02	58.0
Σ_{23}	6.43	0.03	22.70	1.01	1.02	48.1
Σ_{31}	-0.12	-0.87	0.52	1.02	1.05	947.4
Σ_{32}	6.43	0.03	22.70	1.01	1.02	48.1
Σ_{33}	6.62	0.31	22.94	1.01	1.02	56.3
ζ_{11}	0.10	-0.89	0.97	1.00	1.00	2477.7
ζ_{21}	-0.08	-1.11	0.86	1.00	1.00	2458.1
ζ_{31}	-0.05	-1.29	1.16	1.00	1.00	2533.8
ζ_{41}	0.32	-0.87	1.50	1.00	1.00	2673.0
ζ_{51}	0.17	-0.08	0.44	1.00	1.00	2709.0
ζ_{12}	5.33	0.42	10.33	1.02	1.06	245.4
ζ_{22}	3.35	-1.51	8.44	1.02	1.06	507.2
ζ_{32}	4.37	-1.59	10.50	1.01	1.03	615.1
ζ_{42}	2.99	-2.46	8.54	1.02	1.06	550.5
ζ_{52}	0.15	-1.06	1.48	1.01	1.04	577.7
ζ_{13}	3.74	-1.12	8.61	1.01	1.04	233.2
ζ_{23}	2.06	-2.74	7.13	1.01	1.04	460.6
ζ_{33}	2.35	-3.31	8.56	1.01	1.02	657.8
ζ_{43}	1.69	-4.00	8.02	1.01	1.05	513.6

ζ_{53}	0.39	-0.79	1.67	1.01	1.03	639.8
α_1	0.56	0.23	0.93	1.00	1.01	2895.7
α_2	0.74	0.01	1.53	1.00	1.01	2895.1
α_3	0.67	0.42	0.94	1.00	1.00	4150.2
α_4	0.22	-0.05	0.52	1.00	1.00	3000.0
α_5	0.28	-0.32	0.91	1.00	1.00	2973.3
α_6	0.88	0.24	1.57	1.00	1.00	3213.9
α_7	0.47	-0.16	1.18	1.00	1.01	3139.2
α_8	0.55	0.06	1.05	1.00	1.01	3000.0
α_9	0.54	-0.11	1.22	1.00	1.00	3000.0
α_{10}	1.12	0.47	1.90	1.00	1.01	2720.8
α_{11}	0.33	-0.46	1.10	1.00	1.00	2902.7
α_{12}	0.79	0.31	1.32	1.00	1.00	3000.0
α_{13}	0.53	0.13	0.99	1.00	1.00	3000.0
α_{14}	0.87	0.55	1.25	1.00	1.00	3150.3
α_{15}	0.94	0.51	1.45	1.00	1.00	2865.9
α_{16}	0.63	0.10	1.21	1.00	1.00	2717.4
α_{17}	0.99	0.53	1.50	1.00	1.00	2895.3
α_{18}	1.08	0.57	1.63	1.00	1.00	3048.5
α_{19}	0.55	-0.30	1.37	1.00	1.01	2667.8
α_{20}	0.85	0.29	1.50	1.00	1.00	2150.9
α_{21}	1.33	0.97	1.77	1.01	1.02	2830.9
α_{22}	0.10	-0.34	0.59	1.00	1.00	2499.6
α_{23}	0.95	0.29	1.69	1.00	1.01	2856.3
α_{24}	0.76	0.27	1.34	1.00	1.00	3000.0
α_{25}	0.29	-0.16	0.77	1.00	1.01	3000.0
α_{26}	1.05	0.44	1.80	1.00	1.00	3000.0
α_{27}	0.26	-0.45	1.06	1.00	1.00	3002.8
α_{28}	0.11	-0.53	0.75	1.00	1.00	3154.7
α_{29}	0.66	0.00	1.33	1.00	1.00	2797.3
α_{30}	0.69	0.18	1.24	1.00	1.00	2783.5
α_{31}	0.78	0.11	1.53	1.00	1.01	2763.3
α_{32}	0.83	0.24	1.48	1.00	1.01	2734.6
α_{33}	0.52	-0.20	1.30	1.00	1.00	2799.2
α_{34}	0.23	-0.57	1.04	1.00	1.01	3028.5
α_{35}	1.12	0.51	1.84	1.00	1.00	2646.7
α_{36}	0.61	-0.20	1.49	1.00	1.01	2875.4
α_{37}	0.47	-0.07	1.07	1.00	1.00	2876.0
α_{38}	0.86	0.14	1.66	1.00	1.00	2899.2
α_{39}	0.47	0.24	0.70	1.00	1.01	3252.4
α_{40}	0.73	0.44	1.01	1.00	1.00	2684.1
α_{41}	0.92	0.74	1.09	1.00	1.00	3000.0
α_{42}	0.74	0.14	1.43	1.00	1.01	2876.4
α_{43}	1.15	0.79	1.54	1.00	1.00	2764.7
$\beta^{(1)}_1$	5.02	1.93	10.56	1.02	1.07	142.4
$\beta^{(1)}_2$	4.83	1.03	11.52	1.03	1.10	380.8

$\beta^{(1)}_{3}$	5.38	2.81	9.55	1.08	1.22	119.8
$\beta^{(1)}_{4}$	5.63	2.24	11.00	1.11	1.35	58.0
$\beta^{(1)}_{5}$	4.79	0.91	11.14	1.02	1.05	248.7
$\beta^{(1)}_{6}$	5.04	1.02	12.34	1.02	1.03	285.3
$\beta^{(1)}_{7}$	4.78	0.83	11.34	1.01	1.02	372.6
$\beta^{(1)}_{8}$	3.89	0.44	8.56	1.05	1.15	183.6
$\beta^{(1)}_{9}$	6.22	1.79	12.97	1.03	1.04	210.6
$\beta^{(1)}_{10}$	0.62	-4.27	4.44	1.01	1.04	308.9
$\beta^{(1)}_{11}$	4.11	-0.32	11.06	1.01	1.02	308.1
$\beta^{(1)}_{12}$	2.99	0.31	6.16	1.01	1.03	722.2
$\beta^{(1)}_{13}$	4.40	1.41	9.72	1.03	1.04	135.1
$\beta^{(1)}_{14}$	5.72	2.65	10.14	1.02	1.03	162.7
$\beta^{(1)}_{15}$	4.86	1.73	9.76	1.02	1.03	147.5
$\beta^{(1)}_{16}$	4.08	0.47	9.31	1.01	1.02	315.7
$\beta^{(1)}_{17}$	3.16	1.10	5.79	1.00	1.00	991.5
$\beta^{(1)}_{18}$	4.07	0.46	9.59	1.02	1.06	168.6
$\beta^{(1)}_{19}$	3.88	-0.10	11.45	1.02	1.03	428.5
$\beta^{(1)}_{20}$	-0.74	-3.85	1.74	1.02	1.06	343.2
$\beta^{(1)}_{21}$	5.84	2.37	12.59	1.01	1.04	149.3
$\beta^{(1)}_{22}$	1.03	-1.31	3.58	1.03	1.09	298.7
$\beta^{(1)}_{23}$	5.49	0.84	13.49	1.01	1.02	273.6
$\beta^{(1)}_{24}$	2.84	0.33	5.61	1.00	1.00	1064.9
$\beta^{(1)}_{25}$	4.98	1.68	11.37	1.02	1.05	201.3
$\beta^{(1)}_{26}$	6.40	2.06	13.56	1.01	1.01	238.2
$\beta^{(1)}_{27}$	6.31	1.74	13.12	1.01	1.01	157.9
$\beta^{(1)}_{28}$	3.57	0.07	8.57	1.01	1.02	277.4
$\beta^{(1)}_{29}$	3.88	-0.13	9.71	1.03	1.09	296.1
$\beta^{(1)}_{30}$	6.10	2.24	11.36	1.04	1.06	133.8
$\beta^{(1)}_{31}$	3.75	-0.29	10.59	1.03	1.09	349.8
$\beta^{(1)}_{32}$	1.74	-1.23	5.02	1.01	1.05	612.0
$\beta^{(1)}_{33}$	4.44	0.09	10.91	1.02	1.05	357.3
$\beta^{(1)}_{34}$	3.66	-0.93	11.16	1.00	1.01	321.9
$\beta^{(1)}_{35}$	4.82	1.26	10.37	1.02	1.07	226.0
$\beta^{(1)}_{36}$	5.31	0.91	13.17	1.01	1.02	271.3
$\beta^{(1)}_{37}$	4.90	1.63	10.16	1.02	1.07	245.1
$\beta^{(1)}_{38}$	3.99	-0.28	11.14	1.01	1.02	332.3
$\beta^{(1)}_{39}$	6.17	2.79	13.37	1.03	1.04	106.7
$\beta^{(1)}_{40}$	4.00	1.92	6.49	1.01	1.01	579.4
$\beta^{(1)}_{41}$	3.76	2.58	5.19	1.01	1.02	667.3
$\beta^{(1)}_{42}$	5.68	1.62	14.40	1.01	1.02	209.9
$\beta^{(1)}_{43}$	5.61	2.31	11.50	1.00	1.02	97.1
$\beta^{(2)}_{1}$	4.33	1.46	9.80	1.02	1.07	157.5
$\beta^{(2)}_{2}$	3.89	-0.49	10.13	1.02	1.07	320.5
$\beta^{(2)}_{3}$	5.55	2.77	10.16	1.07	1.19	142.5
$\beta^{(2)}_{4}$	5.32	1.93	10.82	1.12	1.38	90.6
$\beta^{(2)}_{5}$	4.20	0.27	10.63	1.02	1.07	220.1

$\beta^{(2)}_{6}$	4.61	0.67	11.97	1.02	1.03	272.1
$\beta^{(2)}_{7}$	3.50	-0.56	9.49	1.00	1.01	256.2
$\beta^{(2)}_{8}$	4.25	1.16	9.07	1.06	1.16	161.3
$\beta^{(2)}_{9}$	4.75	0.24	10.70	1.03	1.04	232.9
$\beta^{(2)}_{10}$	0.28	-4.31	4.21	1.00	1.01	277.5
$\beta^{(2)}_{11}$	3.39	-1.17	10.63	1.01	1.03	274.0
$\beta^{(2)}_{12}$	2.15	-0.61	5.11	1.01	1.04	652.3
$\beta^{(2)}_{13}$	3.85	0.77	9.06	1.03	1.04	187.5
$\beta^{(2)}_{14}$	4.75	1.47	9.00	1.02	1.02	165.2
$\beta^{(2)}_{15}$	4.00	0.82	8.77	1.02	1.03	151.0
$\beta^{(2)}_{16}$	3.57	0.37	8.49	1.01	1.02	317.9
$\beta^{(2)}_{17}$	2.81	0.44	5.45	1.00	1.00	972.8
$\beta^{(2)}_{18}$	3.90	0.22	9.25	1.01	1.05	173.4
$\beta^{(2)}_{19}$	4.56	0.83	12.06	1.02	1.03	432.1
$\beta^{(2)}_{20}$	-0.37	-3.48	2.26	1.00	1.01	324.1
$\beta^{(2)}_{21}$	4.12	0.24	10.44	1.01	1.04	136.0
$\beta^{(2)}_{22}$	1.35	-0.79	3.75	1.03	1.09	367.7
$\beta^{(2)}_{23}$	4.25	-0.32	12.45	1.01	1.01	294.1
$\beta^{(2)}_{24}$	2.01	-0.75	4.89	1.00	1.00	1180.6
$\beta^{(2)}_{25}$	3.75	0.09	9.96	1.02	1.05	188.9
$\beta^{(2)}_{26}$	5.05	0.76	12.07	1.01	1.01	211.6
$\beta^{(2)}_{27}$	5.21	0.73	11.57	1.01	1.01	157.2
$\beta^{(2)}_{28}$	3.65	0.18	8.99	1.01	1.01	252.5
$\beta^{(2)}_{29}$	3.69	-0.10	9.15	1.03	1.09	328.1
$\beta^{(2)}_{30}$	5.28	1.47	10.45	1.04	1.07	162.5
$\beta^{(2)}_{31}$	4.28	0.43	10.91	1.03	1.07	395.1
$\beta^{(2)}_{32}$	1.70	-1.57	4.83	1.01	1.03	520.9
$\beta^{(2)}_{33}$	3.78	-0.46	9.76	1.02	1.05	290.6
$\beta^{(2)}_{34}$	3.39	-0.81	10.62	1.00	1.00	321.5
$\beta^{(2)}_{35}$	4.01	-0.05	9.75	1.02	1.06	272.3
$\beta^{(2)}_{36}$	4.17	-0.21	12.07	1.01	1.02	267.6
$\beta^{(2)}_{37}$	4.53	1.39	9.89	1.03	1.10	213.5
$\beta^{(2)}_{38}$	4.64	0.58	12.09	1.01	1.01	363.1
$\beta^{(2)}_{39}$	5.23	1.90	12.46	1.03	1.05	114.5
$\beta^{(2)}_{40}$	2.97	0.73	5.59	1.00	1.01	626.9
$\beta^{(2)}_{41}$	2.84	1.54	4.31	1.01	1.02	566.3
$\beta^{(2)}_{42}$	4.34	0.09	12.64	1.01	1.02	216.7
$\beta^{(2)}_{43}$	5.38	1.88	11.59	1.01	1.02	81.7

Table S4. Parameters estimates and convergence diagnostics of the MCMC approach used to sample the posterior distribution for the moth case study with real data. The estimated parameters are ρ, Σ , ζ_{kp}, D_s, k_s, m_s, q_s, where $k = 1, \dots, 5$ represents the trait, s$= 1, \dots, 87$ the species, and $p = 1, \dots, 4$ the movement and capture parameters. We show the posterior mean, the 0.025 and 0.975 quantiles, the potential scale

reduction factor (Rhat), the upper 95% confidence limit (95% C.I.) and the effective sample size (n.eff) for each parameter.

Parameter	Mean	0.025 quantile	0.975 quantile	Rhat	95% C.I.	n.eff
ρ	0.13	0.00	0.68	1.02	1.05	695.3
Σ_{11}	5.64	2.16	11.50	1.00	1.02	482.1
Σ_{12}	0.16	-0.69	1.06	1.00	1.00	774.4
Σ_{13}	-2.16	-5.86	0.46	1.02	1.05	480.2
Σ_{14}	3.40	-1.71	10.83	1.07	1.19	217.6
Σ_{21}	0.16	-0.69	1.06	1.00	1.00	774.4
Σ_{22}	0.38	0.16	0.77	1.00	1.01	1332.0
Σ_{23}	-0.11	-0.94	0.81	1.02	1.03	892.9
Σ_{24}	-0.01	-1.53	1.95	1.01	1.03	445.8
Σ_{31}	-2.16	-5.86	0.46	1.02	1.05	480.2
Σ_{32}	-0.11	-0.94	0.81	1.02	1.03	892.9
Σ_{33}	4.78	2.15	9.54	1.03	1.09	558.8
Σ_{34}	5.43	0.69	12.51	1.02	1.06	309.3
Σ_{41}	3.40	-1.71	10.83	1.07	1.19	217.6
Σ_{42}	-0.01	-1.53	1.95	1.01	1.03	445.8
Σ_{43}	5.43	0.69	12.51	1.02	1.06	309.3
Σ_{44}	16.31	5.71	35.91	1.13	1.36	138.8
ζ_{11}	-3.11	-4.90	-1.25	1.02	1.07	411.0
ζ_{21}	-1.11	-4.56	2.05	1.05	1.15	386.1
ζ_{31}	2.03	0.22	3.87	1.01	1.05	567.1
ζ_{41}	1.36	-0.89	3.49	1.02	1.06	648.4
ζ_{51}	-1.10	-2.26	0.06	1.05	1.15	385.7
ζ_{12}	-1.55	-2.15	-0.96	1.01	1.03	1330.4
ζ_{22}	0.61	-0.24	1.42	1.01	1.02	1544.6
ζ_{32}	0.41	-0.19	1.01	1.01	1.02	1444.9
ζ_{42}	-0.57	-1.13	0.12	1.00	1.01	685.6
ζ_{52}	0.01	-0.29	0.31	1.01	1.03	1517.9
ζ_{13}	9.70	8.00	11.53	1.03	1.10	448.9
ζ_{23}	1.57	-1.00	4.65	1.02	1.07	460.0
ζ_{33}	0.64	-1.02	2.40	1.00	1.01	681.4
ζ_{43}	1.40	-0.43	3.30	1.03	1.08	523.2
ζ_{53}	2.07	1.02	3.19	1.01	1.02	386.7
ζ_{14}	-2.36	-4.87	0.42	1.04	1.14	247.6
ζ_{24}	11.19	2.90	21.60	1.19	1.53	48.1
ζ_{34}	5.36	1.90	9.07	1.12	1.37	124.5
ζ_{44}	2.95	-0.57	7.36	1.01	1.04	155.5
ζ_{54}	2.70	0.62	4.94	1.27	1.75	101.3
D_1	-0.23	-5.02	5.25	1.00	1.01	1148.4
D_2	-3.81	-8.84	0.72	1.02	1.06	714.2
D_3	0.58	-3.47	5.02	1.00	1.00	1286.8
D_4	-1.99	-6.71	2.60	1.01	1.03	1117.5
D_5	-4.58	-9.38	-1.07	1.16	1.48	124.8

D_6	-2.44	-7.76	2.19	1.01	1.02	697.4
D_7	-2.13	-7.39	2.87	1.00	1.00	842.3
D_8	0.06	-1.93	2.12	1.00	1.01	2482.4
D_9	-0.88	-5.84	4.25	1.00	1.02	568.2
D_{10}	-2.65	-8.48	2.00	1.03	1.08	443.8
D_{11}	2.05	-2.02	6.24	1.01	1.02	893.3
D_{12}	-0.57	-2.37	1.15	1.00	1.02	1465.9
D_{13}	-2.11	-6.16	1.30	1.02	1.06	918.4
D_{14}	-3.16	-7.91	1.58	1.00	1.01	766.4
D_{15}	-1.16	-6.62	2.05	1.04	1.08	697.6
D_{16}	-4.59	-7.60	-1.98	1.00	1.01	634.5
D_{17}	3.17	0.13	6.84	1.02	1.06	706.0
D_{18}	2.00	-3.33	6.71	1.00	1.01	843.3
D_{19}	-0.82	-4.93	3.58	1.03	1.10	648.6
D_{20}	-1.91	-7.25	3.15	1.00	1.00	700.9
D_{21}	0.46	-4.21	5.50	1.01	1.02	799.2
D_{22}	-1.03	-6.53	3.91	1.04	1.12	682.9
D_{23}	-0.27	-0.96	0.39	1.00	1.00	2835.6
D_{24}	1.85	-1.55	5.92	1.00	1.01	935.6
D_{25}	0.97	-2.13	4.38	1.02	1.06	760.4
D_{26}	0.16	-1.44	2.01	1.00	1.00	1666.7
D_{27}	-4.96	-7.02	-2.98	1.00	1.01	926.4
D_{28}	0.72	-3.62	5.04	1.00	1.00	792.4
D_{29}	-4.17	-5.00	-3.17	1.00	1.00	1281.7
D_{30}	-5.08	-8.84	-2.43	1.08	1.21	386.7
D_{31}	-0.36	-1.07	0.35	1.00	1.00	2747.2
D_{32}	-4.41	-9.43	0.41	1.01	1.04	556.4
D_{33}	-1.78	-7.31	3.29	1.01	1.02	958.1
D_{34}	-4.13	-9.69	1.17	1.04	1.13	508.8
D_{35}	-1.66	-6.31	3.43	1.00	1.01	930.7
D_{36}	2.87	0.01	7.36	1.01	1.02	604.6
D_{37}	1.33	-2.05	5.75	1.01	1.04	539.6
D_{38}	-4.49	-8.78	-0.91	1.03	1.09	1184.6
D_{39}	-2.22	-7.13	2.52	1.01	1.02	1033.8
D_{40}	-2.22	-8.58	2.36	1.01	1.02	512.5
D_{41}	-3.80	-9.39	1.60	1.01	1.03	647.6
D_{42}	1.29	0.43	2.19	1.00	1.00	2752.8
D_{43}	-0.21	-5.47	4.78	1.00	1.00	825.9
D_{44}	-3.34	-5.53	-1.39	1.01	1.03	1428.1

D_{45}	-1.14	-2.55	0.22	1.00	1.01	2287.6
D_{46}	-2.36	-7.16	2.57	1.00	1.00	720.2
D_{47}	-4.55	-9.95	0.36	1.01	1.05	1011.1
D_{48}	0.50	-3.80	5.08	1.00	1.01	1280.3
D_{49}	-0.09	-1.51	1.49	1.02	1.02	1496.6
D_{50}	-1.49	-5.75	3.14	1.01	1.01	700.7
D_{51}	-2.80	-4.66	-1.02	1.00	1.00	1560.1
D_{52}	-5.74	-10.42	-2.18	1.02	1.06	719.4
D_{53}	-1.54	-6.59	3.90	1.03	1.09	636.3
D_{54}	-4.03	-9.47	0.06	1.01	1.02	575.6
D_{55}	-1.91	-7.00	3.38	1.00	1.00	816.6
D_{56}	-3.75	-6.90	-0.68	1.01	1.03	1133.3
D_{57}	-1.42	-6.90	3.32	1.02	1.05	1250.8
D_{58}	-1.86	-7.28	2.99	1.01	1.05	854.6
D_{59}	-2.80	-8.13	2.36	1.00	1.00	637.9
D_{60}	-4.52	-10.01	0.91	1.02	1.07	719.8
D_{61}	0.18	-5.07	5.58	1.01	1.03	1192.3
D_{62}	-5.66	-8.92	-2.77	1.01	1.04	669.6
D_{63}	-2.90	-6.86	1.13	1.01	1.02	862.4
D_{64}	-7.53	-14.17	-1.11	1.05	1.17	531.4
D_{65}	-3.90	-7.79	-0.84	1.01	1.04	1107.3
D_{66}	-0.96	-5.77	3.67	1.01	1.03	833.8
D_{67}	0.83	-3.25	5.15	1.00	1.00	1058.9
D_{68}	-3.56	-6.08	-1.13	1.00	1.01	1395.3
D_{69}	-3.14	-8.64	2.33	1.01	1.02	642.1
D_{70}	-1.32	-6.39	3.72	1.02	1.08	747.6
D_{71}	-1.37	-6.20	3.37	1.01	1.05	814.8
D_{72}	1.33	-2.67	5.89	1.00	1.02	373.3
D_{73}	-0.32	-6.22	4.58	1.01	1.02	994.0
D_{74}	1.66	-2.94	6.33	1.00	1.00	993.7
D_{75}	0.24	-4.30	4.83	1.00	1.00	1288.8
D_{76}	-1.59	-5.26	0.58	1.02	1.04	1164.7
D_{77}	0.99	-4.05	5.65	1.01	1.02	859.7
D_{78}	-2.77	-7.63	1.97	1.00	1.01	977.9
D_{79}	-0.32	-5.89	5.25	1.02	1.05	765.5
D_{80}	0.57	-4.13	5.53	1.00	1.00	898.5
D_{81}	-3.39	-7.43	-0.29	1.02	1.07	1047.7
D_{82}	1.56	-2.88	5.71	1.01	1.02	1121.4
D_{83}	-1.32	-6.00	3.23	1.00	1.01	794.7
D_{84}	-3.72	-8.63	0.90	1.00	1.01	854.9
D_{85}	-1.75	-7.30	3.92	1.02	1.06	699.0
D_{86}	1.63	-3.59	6.75	1.01	1.02	946.8
D_{87}	-1.19	-3.00	-0.03	1.01	1.02	428.7
k_1	-1.72	-2.92	-0.36	1.00	1.01	2248.1
k_2	-1.50	-2.94	-0.14	1.01	1.02	1527.5

k_3	-1.70	-2.78	-0.74	1.00	1.00	1826.1
k_4	-1.73	-3.05	-0.56	1.00	1.01	1609.2
k_5	-1.09	-1.44	-0.78	1.01	1.03	1327.5
k_6	-1.42	-2.79	0.00	1.01	1.03	1868.0
k_7	-1.54	-2.93	-0.18	1.01	1.05	1875.4
k_8	-1.61	-2.49	-0.84	1.00	1.00	2817.7
k_9	-0.84	-2.07	0.42	1.00	1.01	1748.9
k_{10}	-2.08	-3.51	-0.54	1.00	1.01	1571.9
k_{11}	-1.72	-2.53	-0.96	1.00	1.01	2472.7
k_{12}	-1.94	-2.88	-1.19	1.00	1.00	1188.1
k_{13}	-0.90	-1.98	-0.05	1.00	1.00	1282.1
k_{14}	-1.53	-2.81	-0.09	1.00	1.00	1797.2
k_{15}	-1.58	-2.51	-0.78	1.01	1.04	1754.7
k_{16}	-2.01	-2.72	-1.50	1.01	1.03	1555.3
k_{17}	-1.71	-2.69	-0.90	1.01	1.03	966.4
k_{18}	-1.28	-2.45	-0.29	1.00	1.00	1851.8
k_{19}	-2.24	-3.43	-1.10	1.00	1.01	1387.0
k_{20}	-1.50	-2.96	0.00	1.01	1.05	2022.8
k_{21}	-1.69	-2.76	-0.38	1.00	1.01	2046.9
k_{22}	-1.98	-3.43	-0.43	1.02	1.08	1595.2
k_{23}	-2.33	-2.86	-1.88	1.00	1.01	2746.3
k_{24}	-1.01	-1.95	-0.21	1.01	1.02	1299.4
k_{25}	-1.78	-3.19	-0.65	1.00	1.01	914.8
k_{26}	-1.81	-2.72	-1.07	1.00	1.01	1360.9
k_{27}	-2.02	-3.10	-1.23	1.01	1.03	1779.1
k_{28}	-1.65	-2.84	-0.30	1.00	1.01	2063.7
k_{29}	-2.71	-3.10	-2.37	1.00	1.00	2744.0
k_{30}	-1.31	-1.65	-1.01	1.00	1.01	1720.2
k_{31}	-3.46	-4.29	-2.80	1.00	1.00	2379.9
k_{32}	-1.42	-2.80	-0.03	1.01	1.02	1479.7
k_{33}	-1.64	-3.06	-0.18	1.00	1.00	1644.9
k_{34}	-1.06	-2.47	0.28	1.00	1.01	1587.4
k_{35}	-2.04	-3.29	-0.56	1.01	1.02	1851.9
k_{36}	-1.41	-2.28	-0.73	1.00	1.01	1194.3
k_{37}	-1.59	-2.66	-0.68	1.00	1.01	1823.4
k_{38}	-1.31	-2.52	-0.11	1.00	1.00	2484.3
k_{39}	-1.51	-2.90	-0.18	1.01	1.03	1984.6

k_{40}	-1.07	-2.37	0.28	1.01	1.02	1507.9
k_{41}	-0.90	-2.25	0.55	1.02	1.06	2288.6
k_{42}	-2.40	-2.95	-1.97	1.00	1.00	2913.6
k_{43}	-1.57	-2.86	-0.28	1.00	1.01	1566.8
k_{44}	-1.74	-2.19	-1.30	1.01	1.03	2224.4
k_{45}	-1.74	-2.45	-1.20	1.00	1.00	2545.4
k_{46}	-1.54	-2.93	-0.16	1.00	1.01	1879.2
k_{47}	-1.46	-2.82	-0.03	1.00	1.01	2372.7
k_{48}	-1.62	-2.84	-0.32	1.00	1.00	2347.4
k_{49}	-1.95	-2.68	-1.33	1.02	1.04	1637.2
k_{50}	-2.05	-3.38	-0.68	1.00	1.01	1752.2
k_{51}	-1.80	-2.56	-1.19	1.01	1.01	2111.1
k_{52}	-1.13	-1.88	-0.42	1.01	1.02	3000.0
k_{53}	-1.29	-2.80	0.26	1.02	1.07	1251.4
k_{54}	-1.10	-2.29	-0.28	1.01	1.05	860.9
k_{55}	-1.56	-2.93	-0.19	1.01	1.02	1779.7
k_{56}	-1.25	-2.31	-0.30	1.00	1.01	1963.2
k_{57}	-1.09	-2.37	0.17	1.00	1.01	2863.6
k_{58}	-1.99	-3.45	-0.52	1.00	1.01	1419.1
k_{59}	-1.54	-2.90	-0.26	1.01	1.03	1967.7
k_{60}	-1.38	-2.72	-0.06	1.01	1.02	2086.4
k_{61}	-1.71	-2.95	-0.47	1.00	1.00	2144.3
k_{62}	-1.39	-2.05	-0.87	1.00	1.00	2424.6
k_{63}	-1.35	-2.46	-0.25	1.00	1.00	1445.2
k_{64}	-1.51	-3.26	0.22	1.00	1.01	1916.9
k_{65}	-1.72	-2.76	-0.89	1.00	1.01	1870.7
k_{66}	-1.06	-2.36	0.24	1.01	1.03	2074.8
k_{67}	-1.55	-2.76	-0.33	1.00	1.00	1695.2
k_{68}	-1.54	-2.51	-0.80	1.00	1.02	1693.8
k_{69}	-1.46	-2.70	0.04	1.01	1.04	1515.7
k_{70}	-1.52	-2.93	-0.03	1.01	1.02	1731.1
k_{71}	-2.07	-3.36	-0.52	1.01	1.02	1732.9
k_{72}	-1.55	-2.61	-0.62	1.00	1.00	1278.2
k_{73}	-2.37	-3.49	-1.31	1.00	1.02	2428.2
k_{74}	-1.76	-3.04	-0.51	1.00	1.01	2468.8
k_{75}	-1.64	-2.87	-0.32	1.00	1.00	1932.6
k_{76}	-1.76	-2.76	-1.02	1.00	1.00	2008.0
k_{77}	-1.58	-2.81	-0.29	1.00	1.01	1784.1
k_{78}	-1.54	-2.80	-0.20	1.00	1.01	2227.0
k_{79}	-2.07	-3.57	-0.34	1.01	1.02	1526.1
k_{80}	-1.58	-2.85	-0.32	1.00	1.00	2029.6
k_{81}	-1.25	-2.46	-0.27	1.00	1.00	2218.9
k_{82}	-1.17	-2.11	-0.28	1.00	1.00	1620.6
k_{83}	-0.95	-2.15	0.28	1.00	1.01	1702.5
k_{84}	-1.51	-2.81	-0.19	1.01	1.02	2424.1

k_{85}	-1.53	-2.96	-0.15	1.01	1.04	2008.5
k_{86}	-1.75	-3.04	-0.30	1.00	1.01	2143.8
k_{87}	-0.90	-1.33	-0.52	1.00	1.01	1797.5
m_{1}	13.56	9.03	17.85	1.00	1.01	775.8
m_{2}	8.72	4.34	12.50	1.02	1.05	882.3
m_{3}	10.70	8.95	14.14	1.01	1.01	2449.6
m_{4}	9.34	6.11	13.23	1.01	1.02	1202.0
m_{5}	10.05	7.95	14.67	1.19	1.58	100.6
m_{6}	7.92	3.52	12.17	1.01	1.04	999.5
m_{7}	7.04	2.34	11.58	1.01	1.03	910.1
m_{8}	10.93	9.81	12.46	1.00	1.01	2626.0
m_{9}	9.45	4.46	14.27	1.01	1.03	594.9
m_{10}	14.04	9.19	18.88	1.02	1.04	562.3
m_{11}	9.87	8.76	11.94	1.00	1.00	2168.3
m_{12}	10.98	9.94	12.36	1.01	1.03	1207.1
m_{13}	10.16	6.61	14.05	1.00	1.01	1144.3
m_{14}	8.53	4.03	13.23	1.02	1.07	696.9
m_{15}	9.61	7.47	14.72	1.07	1.19	517.8
m_{16}	12.32	10.81	14.87	1.00	1.00	721.6
m_{17}	7.78	4.97	9.50	1.02	1.06	637.1
m_{18}	6.77	2.99	10.34	1.00	1.00	978.7
m_{19}	8.93	4.71	14.20	1.02	1.06	301.1
m_{20}	7.04	2.04	11.73	1.01	1.03	606.1
m_{21}	12.14	8.04	16.35	1.00	1.01	902.3
m_{22}	8.47	3.66	13.23	1.00	1.01	778.1
m_{23}	11.40	11.12	11.73	1.00	1.00	3122.5
m_{24}	6.30	2.74	9.26	1.00	1.01	880.0
m_{25}	7.93	6.36	10.55	1.00	1.00	1309.5
m_{26}	13.40	11.26	17.67	1.00	1.01	756.1
m_{27}	12.44	11.13	14.07	1.00	1.01	961.8
m_{28}	12.12	7.78	15.98	1.01	1.03	1046.4
m_{29}	12.08	11.58	12.66	1.00	1.00	1315.7
m_{30}	13.41	11.41	16.98	1.09	1.24	386.1
m_{31}	11.11	10.93	11.34	1.01	1.02	3249.7
m_{32}	9.12	4.94	13.18	1.01	1.01	755.1
m_{33}	15.43	10.33	20.96	1.01	1.01	710.5
m_{34}	15.81	10.99	21.41	1.01	1.02	625.2

m_{35}	10.79	6.54	15.11	1.01	1.02	775.7
m_{36}	8.62	7.15	10.40	1.00	1.00	1710.2
m_{37}	12.58	11.16	14.55	1.00	1.00	1847.2
m_{38}	16.21	13.08	20.37	1.01	1.03	839.6
m_{39}	8.03	3.13	13.09	1.00	1.02	1067.6
m_{40}	14.22	9.86	18.95	1.01	1.05	567.1
m_{41}	10.66	5.77	16.40	1.04	1.13	758.6
m_{42}	12.23	11.85	12.63	1.00	1.00	2638.1
m_{43}	13.15	8.77	17.89	1.00	1.01	971.3
m_{44}	11.79	10.92	13.34	1.01	1.04	1561.8
m_{45}	7.46	6.56	8.61	1.00	1.00	2229.7
m_{46}	7.96	3.30	12.97	1.02	1.05	1259.6
m_{47}	12.24	7.36	16.83	1.01	1.04	915.5
m_{48}	12.54	8.52	16.85	1.00	1.01	1186.5
m_{49}	15.58	13.39	18.33	1.02	1.06	979.9
m_{50}	12.00	7.40	16.27	1.00	1.01	983.7
m_{51}	10.45	9.56	11.71	1.00	1.00	2006.9
m_{52}	16.23	13.24	20.43	1.01	1.04	681.8
m_{53}	10.57	4.89	15.73	1.05	1.15	573.8
m_{54}	10.36	5.89	14.60	1.03	1.12	392.8
m_{55}	7.66	2.86	12.17	1.01	1.05	883.1
m_{56}	13.45	11.57	16.09	1.01	1.04	1312.7
m_{57}	10.96	6.82	15.61	1.01	1.02	934.3
m_{58}	9.36	5.09	14.24	1.01	1.03	915.8
m_{59}	8.83	3.63	13.44	1.01	1.04	1142.3
m_{60}	12.12	7.94	16.89	1.00	1.01	926.3
m_{61}	11.84	7.36	16.20	1.00	1.00	1433.9
m_{62}	13.92	12.01	16.74	1.01	1.04	739.9
m_{63}	10.95	8.26	14.53	1.01	1.02	1197.4
m_{64}	17.68	11.09	23.77	1.01	1.02	438.0
m_{65}	11.22	8.93	14.44	1.01	1.03	1243.6
m_{66}	9.61	4.78	14.39	1.02	1.06	895.3
m_{67}	12.08	7.77	16.26	1.00	1.00	1122.0
m_{68}	11.40	9.90	13.43	1.00	1.01	1698.4
m_{69}	13.83	9.55	19.13	1.01	1.05	711.4
m_{70}	6.25	1.46	11.86	1.01	1.03	692.8
m_{71}	10.29	6.15	14.54	1.00	1.00	898.5
m_{72}	12.15	9.36	15.59	1.01	1.02	465.2
m_{73}	9.83	6.79	13.69	1.00	1.01	1483.5
m_{74}	9.72	5.39	14.33	1.01	1.03	633.1
m_{75}	12.02	8.00	16.28	1.00	1.00	1208.9
m_{76}	12.01	10.08	16.14	1.02	1.05	967.7
m_{77}	10.83	6.57	14.90	1.01	1.01	648.9
m_{78}	8.75	3.88	13.56	1.01	1.02	981.4

m_{79}	7.46	2.49	12.36	1.02	1.06	696.4
m_{80}	10.77	5.76	14.97	1.00	1.01	769.6
m_{81}	13.84	11.27	17.59	1.02	1.07	1166.9
m_{82}	7.96	4.80	11.37	1.00	1.02	1211.1
m_{83}	10.53	5.87	15.00	1.01	1.03	1063.3
m_{84}	10.74	5.86	15.20	1.01	1.02	1199.0
m_{85}	7.02	2.13	11.71	1.00	1.00	671.5
m_{86}	8.91	3.86	13.82	1.01	1.01	652.5
m_{87}	10.07	9.44	11.97	1.02	1.06	297.1
q_{1}	9.06	0.82	19.00	1.09	1.30	165.7
q_{2}	-5.64	-12.21	-0.95	1.08	1.23	614.8
q_{3}	4.77	0.28	10.65	1.05	1.16	195.5
q_{4}	-1.21	-5.23	3.50	1.00	1.01	901.5
q_{5}	6.88	0.89	17.27	1.12	1.37	77.0
q_{6}	-5.35	-12.86	2.37	1.04	1.11	675.7
q_{7}	-6.57	-14.84	1.45	1.04	1.12	378.9
q_{8}	5.54	0.87	11.18	1.08	1.25	355.7
q_{9}	0.89	-8.40	12.27	1.02	1.07	209.6
q_{10}	5.19	-6.23	16.10	1.11	1.34	195.3
q_{11}	5.16	0.46	12.50	1.05	1.14	193.1
q_{12}	1.52	-1.90	7.39	1.06	1.18	314.5
q_{13}	1.12	-4.39	9.30	1.04	1.12	369.2
q_{14}	-4.92	-12.02	4.99	1.05	1.17	479.2
q_{15}	1.48	-1.22	5.19	1.02	1.06	454.4
q_{16}	0.82	-0.99	4.91	1.09	1.23	253.0
q_{17}	2.80	-2.59	10.36	1.08	1.25	173.7
q_{18}	0.54	-2.98	5.34	1.01	1.02	673.1
q_{19}	-2.39	-6.42	8.61	1.13	1.36	107.1
q_{20}	-6.32	-14.97	4.73	1.04	1.13	235.9
q_{21}	6.97	-1.11	16.25	1.07	1.23	197.2
q_{22}	-4.11	-12.70	8.53	1.06	1.18	505.8
q_{23}	4.87	2.23	8.73	1.04	1.12	230.8
q_{24}	-0.64	-3.83	5.62	1.02	1.05	336.2
q_{25}	-1.48	-3.56	2.60	1.00	1.01	624.9
q_{26}	9.26	1.34	17.97	1.10	1.30	167.5
q_{27}	-1.45	-2.24	-0.48	1.01	1.03	1771.5
q_{28}	7.22	-2.47	16.42	1.05	1.13	123.6
q_{29}	-0.20	-0.70	0.61	1.02	1.03	1378.4
q_{30}	3.01	0.71	6.19	1.06	1.19	304.5
q_{31}	4.03	2.26	6.56	1.01	1.04	934.5
q_{32}	-5.82	-12.18	-1.33	1.02	1.05	653.1
q_{33}	10.55	0.73	20.14	1.15	1.44	158.2
q_{34}	10.05	0.21	21.70	1.14	1.43	192.9
q_{35}	0.02	-8.12	9.44	1.01	1.05	678.9
q_{36}	3.63	-1.77	11.85	1.03	1.07	150.1
q_{37}	8.10	-0.11	16.15	1.03	1.07	181.0

q_{38}	9.93	2.85	18.70	1.17	1.49	172.6
q_{39}	-4.52	-12.22	3.88	1.02	1.07	715.6
q_{40}	15.48	4.18	27.59	1.03	1.10	108.0
q_{41}	8.27	-2.31	19.05	1.23	1.66	158.9
q_{42}	8.25	4.23	13.11	1.13	1.41	164.6
q_{43}	7.95	-1.89	18.07	1.07	1.21	225.1
q_{44}	9.60	1.88	19.84	1.20	1.57	61.6
q_{45}	-2.54	-3.31	-1.73	1.00	1.00	2836.1
q_{46}	-4.92	-13.09	3.29	1.03	1.09	489.3
q_{47}	0.67	-8.14	9.49	1.02	1.08	529.9
q_{48}	7.73	-1.61	16.20	1.06	1.20	222.9
q_{49}	12.89	6.84	20.90	1.16	1.53	105.3
q_{50}	2.75	-6.56	12.75	1.01	1.02	393.8
q_{51}	-1.70	-2.97	0.20	1.01	1.03	1414.4
q_{52}	16.40	7.45	27.49	1.02	1.05	76.1
q_{53}	8.89	-6.76	23.25	1.24	1.66	114.4
q_{54}	7.14	-2.84	19.64	1.19	1.54	67.8
q_{55}	-4.91	-13.24	3.23	1.03	1.12	553.4
q_{56}	5.21	1.16	10.47	1.10	1.31	222.5
q_{57}	3.77	-4.04	12.44	1.02	1.06	417.6
q_{58}	-3.47	-10.90	8.99	1.04	1.14	378.4
q_{59}	-3.73	-11.61	5.17	1.02	1.05	364.7
q_{60}	0.36	-8.30	10.03	1.02	1.07	498.9
q_{61}	6.00	-2.75	15.66	1.03	1.09	418.9
q_{62}	5.14	0.73	11.77	1.23	1.69	114.9
q_{63}	-0.03	-3.92	5.17	1.03	1.05	662.7
q_{64}	7.85	-3.49	20.71	1.17	1.51	154.0
q_{65}	-0.68	-2.81	3.20	1.02	1.03	776.1
q_{66}	1.29	-7.09	12.41	1.02	1.08	387.7
q_{67}	7.20	-3.29	16.85	1.04	1.10	198.3
q_{68}	-0.12	-1.90	2.91	1.01	1.02	955.0
q_{69}	13.70	3.08	26.59	1.08	1.26	109.8
q_{70}	-7.14	-15.53	3.94	1.12	1.35	267.7
q_{71}	-0.75	-9.18	7.91	1.02	1.05	456.9
q_{72}	7.63	-2.66	15.81	1.05	1.15	126.3
q_{73}	0.63	-3.86	7.37	1.00	1.00	887.7
q_{74}	3.72	-4.97	12.69	1.03	1.06	337.1
q_{75}	6.42	-3.51	16.10	1.02	1.08	311.0
q_{76}	12.13	3.46	23.01	1.12	1.36	63.4
q_{77}	4.87	-4.81	14.44	1.01	1.03	208.4
q_{78}	-3.98	-11.87	3.54	1.04	1.10	637.0
q_{79}	-4.99	-13.72	9.11	1.08	1.25	339.9
q_{80}	4.11	-6.46	14.98	1.01	1.03	274.9
q_{81}	4.91	-0.71	11.17	1.04	1.13	240.5
q_{82}	2.40	-3.06	9.55	1.01	1.03	475.7
q_{83}	2.85	-6.59	12.38	1.05	1.16	380.1

q_{84}	-1.10	-9.44	7.93	1.01	1.03	868.7
q_{85}	-6.15	-14.81	2.39	1.04	1.13	343.6
q_{86}	1.97	-7.44	11.84	1.01	1.01	320.8
q_{87}	2.44	0.04	6.08	1.04	1.12	236.0

References

Gelman, A., Rubin, D.B., 1992. Inference from iterative simulation using multiple sequences. Stat. Sci. 7, 457–511. doi:10.16373/j.cnki.ahr.150049

Plummer, M., Best, N., Cowles, K., Vines, K., 2006. CODA: Convergence Diagnosis and Output Analysis for MCMC. R News 6, 7–11.
